# Guideline adherence in German routine care of children and adolescents with ADHD: an observational study

**DOI:** 10.1007/s00787-020-01559-8

**Published:** 2020-05-28

**Authors:** Kristina Mücke, Julia Plück, Susanne Steinhauser, Martin Hellmich, Kristin Scholz, Astrid Sonneck, Lisa Winkler, Manfred Döpfner

**Affiliations:** 1 Department of Psychiatry, Psychosomatics and Psychotherapy in Childhood and Adolescence, Medical Faculty, University of Cologne, Cologne, Germany; 2 Faculty of Medicine, Institute of Medical Statistics and Computational Biology, University of Cologne, Cologne, Germany; 3 University Hospital Cologne, University of Cologne, Cologne, Germany; 4 School of Child and Adolescent Cognitive Behavior Therapy, University Hospital of Cologne, Cologne, Germany

**Keywords:** ADHD, Guidelines, Adherence, Children and adolescents

## Abstract

Although guidelines for the assessment and treatment of mental disorders in childhood and adolescence have been available in Germany for several years, there are barely any data on adherence to guidelines in national routine care. Therefore, the study aimed at a nationwide evaluation of guideline adherence (GA) for the assessment and treatment of children and adolescents with attention-deficit/hyperactivity disorder (ADHD) in German routine care in various groups of health care providers (HCPs). Besides a detailed description of GA, the study focused on examining possible differences between professional groups. Furthermore, data based on global self-reports of clinicians were compared with ratings of documented care in individual patients. Protocols of 73 clinicians regarding their handling of ADHD in routine care for 167 patients were rated according to German guideline recommendations for ADHD care. GA was measured as the proportion of components fulfilled in each individual patient as documented by the HCP. The results were compared to a preceding interview with clinicians regarding their GA. Multilevel models were constructed to detect differences in GA between professional groups. Based on mandatory guideline components, adherence rates of 38.9–72.7% were found and classified as moderate (33.3% < GA ≤ 66.6%) to high (GA > 66.6%). The comparison of the GA between the professional groups generally yielded only small differences. Correlations between GA reported globally by the HCPs and GA documented and rated for individual cases were low. Overall, most rates of GA for ADHD in German routine care lay within a moderate range. Targets for enhancement of GA may be the involvement of teachers and schools in the treatment process, the implementation of psychoeducational methods in general, as well as a careful examination of patients, including monitoring of treatment effects during titration trials. The development of further strategies to monitor the quality of ADHD routine care is needed.

**Electronic supplementary material:**

The online version of this article (10.1007/s00787-020-01559-8) contains supplementary material, which is available to authorized users.

## Introduction

National guidelines for the assessment and treatment of children and adolescents with attention-deficit/hyperactivity disorder (ADHD) have been developed in many countries (e.g. [1–5]), including Germany [6, 7]. Guidelines have also been published on a European level [8–10]. Moreover, several recommendations and practice parameters for the assessment and treatment of ADHD have been released (e.g. [11–15]). Based on guidelines of the German Association for Child and Adolescent Psychiatry, Psychosomatics and Psychotherapy [6], the German Association for Paediatrics [7] and practice parameters for the assessment and treatment of ADHD [15], the German ADHD network developed a unified practice protocol [16]. In 2018, new evidence and consensus-based German guidelines were released [17]. The main modification of these new guidelines lies in a slight shift in the indication for pharmacological interventions compared to psychological interventions: while the previous guidelines recommended pharmacotherapy after initial psychoeducation for children older than 5 years with severe ADHD, the latest guidelines also recommend pharmacotherapy or psychotherapy for patients (older than 5 years) with moderate ADHD.

Despite this huge engagement in developing guidelines for the assessment and treatment of ADHD over the last 20 years, the extent of their implementation in routine care within outpatient treatment still remains fairly unknown. Previous studies—almost all of them conducted in the United States—can be distinguished regarding data collection. Several studies used global reports of health care providers (HCPs) to analyse the quality of ADHD care, usually finding modest to high adherence to guidelines (e.g. [18–20]). For example, in a survey with 723 primary care physicians in Michigan, 61.1% reported incorporating guidelines into their practice [20]. A national survey by Chan and co-workers [18] found that 70.5% of *n* = 861 primary care paediatricians and family physicians reported using parent ratings, and 70.2% used teacher ratings during ADHD assessment. More recently, a study by McElligott and co-workers [21] with *n* = 42 paediatricians in South Carolina indicated that the majority adhered to the guidelines for ADHD care regarding the use of a validated screening tool (97.6%), stimulants as first-line agents (100%), and appropriate follow-up after initiation of medication (97.6%).

Retrospective chart reviews revealed lower rates of guideline adherence (GA). For example, Epstein and co-workers [19] examined *n* = 1594 patient charts of 50 paediatric practices and found that paediatricians documented the use of standardised parent and teacher ratings of ADHD during assessment in 56.7 and 55.5% of cases, respectively. Concerning pharmacotherapy, 47.4% of the included cases had at least one visit or telephone contact during the first month after prescription. Additionally, only in a minority of cases were parent (10.8%) or teacher ratings (7.5%) used to monitor treatment response. Gordon et al. [22] compared the results of global self-reports of clinicians with retrospective chart reviews and found evidence that either clinicians tend to overestimate their adherence or that the lower rates obtained through chart review may reflect under-documentation.

Parent reports on assessment and treatment conducted by the physician also revealed rather low GA rates. A study using data from the German health insurance company Gmünder Ersatzkasse (GEK) [23] analysed the reports of approximately 2300 parents of children with ADHD treated with medication. According to parent reports, information from teachers was obtained during assessment in 52.1% of cases. Regarding pharmacotherapy, almost half of the patients visited their doctor’s office only once during the first month after prescription. The use of parent ratings during titration was reported in 27.6% of cases and the use of teacher ratings during titration was reported in 20.9% of cases. Furthermore, the study indicated that multimodal treatment—although recommended by the guidelines—is not yet sufficiently implemented in routine care: 94.9% of parents received information about ADHD, whereas only 73.9% reported receiving consultations regarding parenting and the handling of problematic situations. Preschool or school teachers received information or advice in 12.0% of cases, and parent training or family therapy was applied in 19.2 and 6.8%, respectively. Child behaviour therapy was reported by the parents in 27.4% of cases, cognitive therapy in 18.2%, and social skills training in 8.4%. However, since these results are based exclusively on information provided by the parents, their validity may also be questionable. Overall, global reports of HCPs demonstrate acceptable to good GA, while GA based on retrospective chart review and parent reports is substantially lower. This difference may be explained by a tendency of HCPs’ global reports to overestimate GA, as well as a tendency of parents to underestimate GA, since they may not be aware of all measures of the HCP to attain GA. Therefore, the current study also aims to examine the effects of methods and reporting source used in the assessment of GA.

Regardless of the strategy of data collection, previous studies most frequently examined the practice patterns of primary care paediatricians or family physicians. This may explain the fact that these surveys mainly focused on GA during assessment and/or pharmacological treatment, whereas adherence regarding psychoeducation or psychological treatment was rarely analysed. Indeed, while most of the studies mentioned at least the percentage of cases receiving recommendations for psychosocial treatment (e.g. parent training, behaviour therapy), knowledge about process quality for these interventions is still lacking. Moreover, the studies published to date have been limited to specific professional groups of HCPs.

Therefore, the current study was conducted to answer the following research questions:

1. What is the overall degree of GA regarding assessment, treatment indication, psychoeducation, pharmacotherapy and psychotherapy of children and adolescents with ADHD in routine care in Germany?
2. Are there any differences in GA between the main professional groups of HCPs (paediatricians, child and adolescent psychiatrists, child and adolescent psychotherapists) who are involved in outpatient care of children and adolescents with ADHD in Germany?
3. Do rates of GA depend on the method of data collection (global report of HCPs vs. independent ratings of HCP-documented individual care processes?)

## Methods

The current analysis is part of a three-phase study [ImLeiV-ADHS: Implementierung leitlinienorientierter Versorgung von Kindern und Jugendlichen mit ADHS (Implementation of guideline-oriented care of children and adolescents with ADHD)] concerning the quality of assessment and treatment of children and adolescents with ADHD in German routine care. The study was funded by the German Federal Ministry of Health (Bundesministerium für Gesundheit—BMG). The conduct of the trial was approved by the Ethics Committee of the Medical Faculty of the University of Cologne. The trial was registered at the German Clinical Trials Register (DRKS00013489, Universal Trial Number: U1111-1205-6338). The research was undertaken with the understanding and written consent of HCPs, patients, and parents or guardians.

### Recruitment

HCPs were recruited nationwide from January 2014 through April 2015. A total of approximately *N* = 2615 outpatient units and practices of six different types of HCPs were addressed: the clinical directors of all social paediatric centres (*n* = 141), all departments of child and adolescent psychiatry (*n* = 155) as well as all schools for child and adolescent psychotherapy (*n* = 55) with outpatient units registered in Germany were contacted. To establish contacts with paediatricians, child and adolescent psychiatrists as well as all child and adolescent psychotherapists working in practices, random samples from the 2014 membership lists of the National Association of Statutory Health Insurance Physicians (Kassenärztliche Bundesvereinigung, KBV) were selected, resulting in a total random sample of *n* = 2264 HCPs in practices. From the total *N* = 2615 HCPs contacted, *n* = 695 (26.6%) responded, of whom *n* = 452 (17.3%) were willing to participate in the first phase of the study consisting of a structured interview concerning their usual practice in ADHD care. All questions were aimed at guideline components and used equivalent wording: “In how many of your patients with ADHD do you conduct… [specific guideline component]?” [rating: 0 = “almost none”, 1 = “up to 25%”, 2 = “up to 50%”, 3 = “up to 75%”, 4 = “up to 100%” (… of all cases)]. Overall, *n* = 363 (13.9%) HCPs completed the interview. A subsample (*n* = 275; 10.5%) was analysed by Sonneck, Plück and co-workers [24], who found a high level of GA overall. Adherence to most of the defined diagnostic and therapeutic interventions was reported on average for 75.0–100% of all ADHD patients. Participating HCPs were representative of the entire group of initially contacted HCPs concerning the sociodemographic variables which were available, with one exception: HCPs from the outpatient units of schools for psychotherapy were more likely to be men and from the Western part of Germany and less likely to be from Southern or Northern Germany compared to the entire group of initially invited participants. The remaining *n* = 88 HCPs were enrolled in a subsequent recruitment phase to increase the sample size for an ensuing second phase of the study (observation of individual patients).

**Fig. 1:**
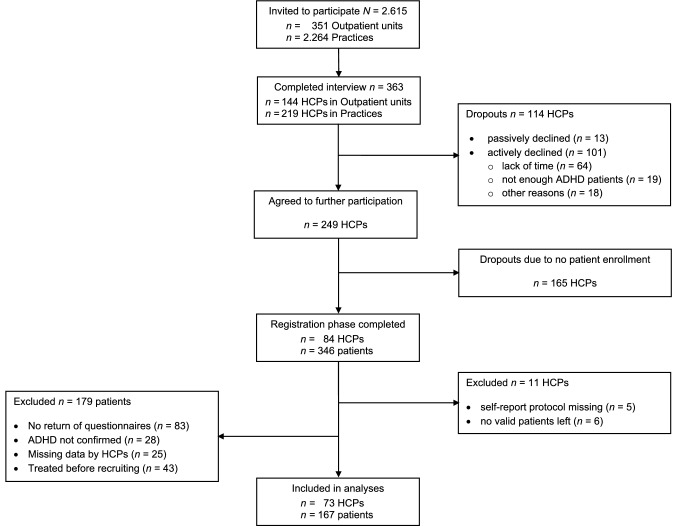
Flow of participants through the study

The interview was a precondition for participation in the second study phase, and *n* = 249 (9.5%) of the *n* = 363 HCPs who completed the interview in phase 1 agreed to participate in phase 2. These HCPs were asked to recruit all eligible patients aged between 6;0 and 17;11 years over a period of 3 months. Of interest were children and adolescents who were referred to the HCPs with an ADHD-related problem for the first time. Further inclusion criteria were as follows: according to the clinical impression, the patients should have no autism spectrum disorder, no acute indication for inpatient treatment and no signs of mental retardation. HCPs verified inclusion criteria based on their clinical judgement. The final sample of *n* = 167 patients was recruited by *n* = 73 HCPs (29.3% of those who initially agreed to participate). All of these patients received an ADHD diagnosis according to ICD-10/DSM-5 criteria based on the clinical judgement of the HCPs (Fig. 1).

### Participants

Nearly half of the HCPs (*n* = 34) included one patient, 23.3% (*n* = 17) two patients and the remaining 30.1% (*n* = 22) three to nine patients (only one HCP recruited the maximum of nine patients). The mean age of the *n* = 167 patients enrolled by the HCPs was *M* = 8.6 years (SD = 2.2), and 72.5% (*n* = 121) were boys. At the start of treatment, the majority of patients (86.8%) showed at least “distinct” symptoms of ADHD and 64.7% of patients showed symptoms of ODD according to parent ratings. Further information about the sample is provided in Table 1.

**Table 1:** Characteristics of HCPs (*n* = 73) and families treated (*n* = 167)

HCP-related characteristics				
Age (years)	*M* (SD), [range]	48.84	(9.51)	[28–66]
Sex (male)	*n* (%)	30	(41.10)	
Qualification completed^a^	*n* (%)	61	(83.56)	
Time in current position (years)	*M* (SD), [range]	10.30	(7.74)	[0–31]
ADHD expertise (years)	*M* (SD), [range]	13.97	(7.72)	[1–34]
Authorisation for pharmacotherapy^b^	*n* (%)	51	(69.86)	
Authorisation for psychotherapy^c^	*n* (%)	48	(65.75)	
Further ADHD training	*n* (%)	36	(49.32)	
Additional ADHD contract^d^	*n* (%)	28	(38.36)	
Family-related characteristics				
Patient				
Age (years)	*M* (SD), [range]	8.63	(2.16)	[6–16]
Sex (male)	*n* (%)	121	(72.46)	
ADHD pre-score	*M* (SD), [range]	1.56	(0.54)	[0.25–3.00]
Parent				
Age (years)	*M* (SD), [range]	39.25	(6.61)	[24–67]
Professional degree^e^	*n* (%)	108	(64.67)	
Academic degree	*n* (%)	36	(21.56)	

*HCPs* health care providers

^a^Specialised medical certificate (paediatrician or child and adolescent psychiatrist) or licence to practise (child and adolescent psychotherapist)

^b^Treatment authorisation for pharmacotherapy: paediatricians and psychiatrists

^c^Treatment authorisation for psychotherapy: psychiatrists and psychotherapists

^d^Participation in programmes offered by several German health insurance companies to support intensified coordination between different HCPs

^e^School-leaving exams PLUS additional professional qualification

### Measures

Both instruments—the interview (first phase, [24] already mentioned in the recruitment section) as well as the care-process protocol (second phase, focused here)—were specifically developed for this study, based on the recommendations of the German ADHD network [16], which combine the current German guidelines for diagnosis and treatment of ADHD. Three months (*T*_M3_) and 6 months (*T*_M6_) after treatment initiation, HCPs retrospectively documented the applied assessment and treatment components for each patient in the care-process protocol (Online Resource 1). This comprised six sections: (1) pre-treatment by other HCPs, (2) assessment, (3) treatment indication, (4) psychoeducation, (5) pharmacotherapy and (6) psychotherapy. Each section included several questions in open format. Besides treatment indication, the other sections addressed questions about the specific interventions carried out in the care process and with whom they were realised (i.e. patient, parents, teachers). In the treatment indication section, HCPs documented whether they considered psychoeducation, pharmacotherapy and/or psychotherapy to be indicated for treatment (not/primary/later). The section for pharmacotherapy recorded information about the specific assessment, and then selection of the drug, titration and long-term medication. HCPs could indicate whether an intervention had been conducted by them personally or by colleagues/co-workers. Moreover, reports about recent assessment results (e.g. intelligence test) or records of medication trials from external pre-treatments were included in the data evaluation.

After prior training and under continuous supervision, GA was rated by one of three research assistants again with reference to the unified practice protocol of the German ADHD network [16]. Rating criteria for each guideline component were operationalised in a rating manual (1 “adherence”; 0 “no adherence”). More information can be found in the electronic supplement of this article (Online Resource 2). In the resulting dataset, information from the two assessment points (*T*_M3_/*T*_M6_) was merged. Five different GA indices were obtained, with three profession-independent sections for assessment (AS = 16 components), treatment indication (TI = 3 components), and psychoeducation (PE = 18 components), and a further two profession-dependent sections for pharmacotherapy (PH = 21 components; physicians only) and psychotherapy (PT = 12 components; child and adolescent psychiatrists and psychotherapists). These five GA indices contained components being mandatory as well as components that are recommended as good clinical practice (hereinafter referred to as “all”). Additionally, GA indices solely comprising a mandatory standard as defined by guidelines were calculated (AS_MAN_ = 8, PE_MAN_ = 2, PH_MAN_ = 10, PT_MAN_ = 4 components). No mandatory standard for treatment indication was computed. In this section of the protocol, HCPs solely documented which of the treatment options (psychoeducation, pharmacotherapy and/or psychotherapy) in which order (primarily or later) they did or did not indicate. Furthermore, the main alteration in the German guidelines in 2018, according to which the primary indication for pharmacotherapy was extended to patients with moderate (as opposed to previously only severe) ADHD symptoms, could also be accounted for by calculating two different indices for treatment indication (Old = TI_OLD_ and New = TI_NEW_). For comparability, all of the indices mentioned above were calculated as the proportion of the possible maximum of adherence (Σ_components_ × 100/*n*_components_) within each area. An overview of GA components (mandatory and all) is provided in the electronic supplement of this article (Online Resource 3, Tables S1–S5).

## Analyses

Several analyses were conducted to determine the representativeness of the sample: differences between the two recruitment waves (*n* = 275 vs. *n* = 88) as well as between HCPs participating in the initial interview only (*n* = 290) vs. HCPs who also included patients (*n* = 73) were tested. Interval-scaled variables (GA indices, age, time in current position and ADHD expertise) were compared using *t* tests. Rates on dichotomous variables (sex, qualification for current position, further ADHD training and additional ADHD contract) were compared using binomial tests. For the section of treatment indication, Mann–Whitney *U* tests were conducted to test for item-wise differences. Moreover, we calculated Pearson correlations of corresponding GA scores from the interview and GA indices from the ratings of documented care within the group of HCPs who participated in both phases (*n* = 73).

To assess the inter-rater reliability of the ratings of GA components following the manual described above, 20.0% of all protocols were evaluated by all three raters. Intraclass correlation coefficients (ICC) were calculated based on the summarised, dichotomously coded components for each rating section. The resulting ICCs ranged from 0.74 ≤ *r* ≤ 1.00, and can thus be considered as satisfactory [25].

The dataset of the second study phase had a hierarchical structure, as patients (Level 1) were nested within HCPs (Level 2). Therefore, data for patients of each HCP in addition were aggregated to a single mean score. These Level 2 variables were used to calculate correlations between interview and documentation as well as to legitimise subsequent sampling of the six types of HCPs. All of these statistical analyses were conducted using SPSS 25 [26]. As no major deviations between values of Level 1 and Level 2 were found, all results concerning GA for the second phase are shown on the patient level only. The results indicate the mean percentages of fulfilled components according to the documented care of patients as rated by research assistants. These rates were calculated for all GA indices (i.e. mandatory, all, TI_OLD_, TI_NEW_) and further classified with values ranging from 0.0 to 33.3% defined as “low”, 33.4–66.6% as “moderate” and 66.7–100.0% as “high”. In addition, it was also determined whether “at least one” of the mandatory components for each section had been documented by the HCPs, as many of the mandatory components per section were thematically linked to each other (e.g. assessment) and the open format of the protocol required very detailed documentation.

Finally, the GA indices including all components were analysed to identify differences between the professional groups concerning the quality of ADHD care as documented for their patients. To account for the clustering by HCP, separate linear mixed models for each GA index (outcome variable) were constructed using REML estimations [27]. Affiliation with one of the three professional groups was introduced as a fixed factor. The Kenward-Roger approximation was used due to the small sample size [28]. Thus, model specifications for these analyses were defined as follows: fixed effect for professional group and random intercept for HCPs. Note that as the TI indices consisted of only three components, estimates might be imprecise. Limited by equal restrictions, another model was built, treating the two TI indices as repeated measurements to detect differences between TI_OLD_ and TI_NEW_ for the total sample. Following this, the fixed effect for professional group was left out, while the other model specifications remained the same. No model was built for PT due to small group sizes. ICCs were calculated to assess the proportion of the total variance of the outcome variable (GA) explained by HCP clustering. Stata 14.2 software [29] was used for these analyses.

## Results

### Representativeness

The sample of the *n* = 73 HCPs who completed both phases comprised a significantly greater proportion of women (*p* = 0.020), of HCPs who were still in training (*p* ≤ 0.001), and of HCPs who were participating in additional ADHD contracts with health insurance companies (*p* ≤ 0.001) compared to the *n* = 290 HCPs who participated in the first phase only (Online Resource 3, Table S6). ADHD contracts provide additional financial support for increasing coordination between different HCPs, especially paediatricians, child and adolescent psychiatrists, and child and adolescent psychotherapists. No significant differences concerning the globally reported GA of the HCPs (interview) were found when comparing the first wave of *n* = 275 HCPs with the subsequently recruited *n* = 88 HCPs, or when comparing the *n* = 290 HCPs who participated in first phase only with the *n* = 73 HCPs who completed both phases (Online Resource 3, Table S7).

### Guideline adherence (GA)

To increase the power for the subsequent analyses, the original six types of HCPs were subsumed into three types based on profession: social-paediatric centres were grouped together with paediatric practices (1 = paediatricians), outpatient units of departments of child and adolescent psychiatry were grouped together with child and adolescent psychiatric practices (2 = psychiatrists), and outpatient units of schools for child and adolescent psychotherapy were combined with child and adolescent psychotherapy practices (3 = psychotherapists). There were no significant differences between these merged groups concerning the calculated GA indices (Online Resource 3, Table S8). Thus, 25 paediatricians with 50 patients [mean *n*_patients_ per paediatrician = 2.0 (SD = 1.5)], 26 psychiatrists with 81 patients [mean *n*_patients_ per psychiatrist = 3.1 (SD = 2.2)] and 22 psychotherapists with 36 patients [mean *n*_patients_ per psychotherapist = 1.6 (SD = 1.1)] were included in the subsequent analyses. HCPs documented an average of 11 (SD = 7.6) office visits per patient during the 6-month period, with a minimum of 1 and maximum of 35 contacts. Individual visits lasted between 10 and 132 min (*M* = 53.5; SD = 16.9). Further information about general treatment characteristics is provided in the electronic supplement (Online Resource 3, Table S9).

Table 2 shows the GA indices for assessment and treatment across all 167 patients based on the documentations of all HCPs (overall) as well as for each professional group. These indices indicate the mean percentage of recommendations followed by the HCPs according to their documentation for each patient.

**Table 2:** Comparison of GA indices: total of components (all) vs. mandatory standard (mandatory)

GA indices^a^	Components^b^	Overall (*n* = 167 patients)			Paediatricians (*n* = 50 patients)			Psychiatrists (*n* = 81 patients)			Psychotherapists (*n* = 36 patients)		
		*M*	SD	Range	*M*	SD	Range	*M*	SD	Range	*M*	SD	Range
Assessment (AS)	Mandatory	51.50	21.64	00.00–100.00	57.50	20.82	12.50–87.50	53.24	18.20	00.00–100.00	39.24	25.38	00.00–75.00
	All	59.36	17.74	00.00–100.00	62.00	17.35	18.75–87.50	60.12	15.20	18.75–100.00	54.00	22.42	00.00–87.50
Treatment indication (TI)^c^	Old	86.43	19.07	00.00–100.00	84.67	21.52	00.00–100.00	87.65	18.59	33.33–100.00	86.11	16.67	66.67–100.00
	New	97.21	12.36	33.33–100.00	94.67	15.59	33.33–100.00	97.53	12.67	33.33–100.00	100.00	0.00	100.00–100.00
Psychoeducation (PE)	Mandatory	38.92	41.58	00.00–100.00	30.00	37.80	00.00–100.00	46.30	43.86	00.00–100.00	34.72	39.31	00.00–100.00
	All	21.19	14.52	00.00–61.11	16.90	13.27	00.00–61.11	22.02	15.30	00.00–61.11	25.31	13.18	00.00–50.00
Pharmacotherapy (PH)	Mandatory	72.67^d,e^	19.70	30.00–100.00	72.50^d^	19.43	40.00–100.00	72.80^e^	20.31	30.00–100.00	n/a		
	All	51.01^d,e^	14.56	23.81–76.19	51.67^d^	14.61	23.81–76.19	50.48^e^	12.26	23.81–76.19	n/a		
Psychotherapy (PT)	Mandatory	43.59^f,g^	18.78	00.00–100.00	n/a			37.50^f^	34.46	00.00–100.00	44.70^g^	15.00	25.00–75.00
	All	37.90^f,g^	13.38	08.33–75.00	n/a			24.13^f^	5.69	16.67–33.33	40.40^g^	12.86	08.33–75.00

*n/a* not available

^a^Guideline adherence indices: mean percentage of components fulfilled according to documented care of patients as rated by research assistants

^b^Detailed information about specific components included in mandatory vs. all can be found in the electronic supplement (S1–S5)

^c^Two different indices for TI were calculated due to a slight change in the guidelines; as both indices already consisted of only three components with one for each treatment option, no mandatory standard was defined

^d^Results refer to *n* = 20 patients personally treated by *n* = 13 paediatricians

^e^Results refer to *n* = 25 patients personally treated by *n* = 17 psychiatrists

^f^Results refer to *n* = 6 patients personally treated by *n* = 4 psychiatrists

^g^Results refer to *n* = 33 patients personally treated by *n* = 21 psychotherapists

The index consisting of the mandatory components for assessment (i.e. clinical interview with parents regarding ADHD symptoms, coexisting conditions, developmental milestones, ADHD course, risk factors, current developmental level, clinical interview with patient and physical examination) averaged 51.5%; the mean for all components lay at 59.4% (Table 2). Fulfilment rates of the six mandatory components concerning the clinical interview with parents ranged from 29.3% for interviewing about the course of ADHD to 64.7% for assessing relevant ADHD symptoms. Active involvement of patients in the initial assessment process was specified for 83.2% of patients and the realisation of a physical examination was documented in 66.5% (Online Resource 3, Table S1). At least one of the eight mandatory components was mentioned in 96.4% of all cases. Optional components were applied in 34.7% (clinical interview with others, e.g. teachers) to 90.4% (use of psychological tests for patient) of cases. HCPs documented the use of ADHD rating scales with parents in 80.8% of cases and with teachers in 60.5%. Rating scales to assess coexisting conditions were used with parents in 60.5% of cases and with teachers in 43.7%. No significant differences in GA were found between the three professional groups with respect to AS (Table 3).

**Table 3:** Results from mixed modeling using professional group to predict guideline adherence in ADHD care

GA indices^b^	Paediatricians (*n* = 50 patients)		Psychiatrists (*n* = 81 patients)		Psychotherapists (*n* = 36 patients)		ICC^a^
	EMM	CI (95%)	EMM	CI (95%)	EMM	CI (95%)	%
Assessment (AS)	61.34	54.71–67.96	58.66	52.54–64.78	55.40	48.17–62.63	55.01
Treatment indication_old_ (TI_OLD_)	83.86	78.06–89.65	86.73	81.92–91.53	85.91	79.28–92.54	10.05
Treatment indication_NEW_ (TI_NEW_)	92.52^e^	87.67–97.37	96.79^e^	92.29–101.30	100.00^e^	94.73–105.27	59.16
Psychoeducation (PE)	14.58^e^	9.48–19.69	20.95^e^	16.25–25.66	26.58^e^	21.01–32.16	53.61
Pharmacotherapy (PH)	51.85^c^	44.33–59.38	50.41^d^	43.78–57.04	n/a	n/a	47.73

*EMM* estimated marginal mean, *n/a* not available

^a^Intraclass Correlation Coefficients: proportion of variance explained by the nested effect

^b^Guideline Adherence Indices: mean percentage of components fulfilled according to documented care of patients as rated by research assistants

^c^Results refer to *n* = 20 patients personally treated by *n* = 13 paediatricians

^d^Results refer to *n* = 25 patients personally treated by *n* = 17 psychiatrists

^e^Groups with different numbers in index differ significantly (at least *p* ≤ 0.05)

The mean percentage of adhering the recommendations for treatment indication for psychoeducation (should be given in all cases), pharmacotherapy (should be given in severe ADHD [old guidelines] or in moderate to severe ADHD [new guidelines]) or psychotherapy (should be given in mild to moderate ADHD) based on the criteria of the old guidelines averaged 86.4%. The index based on the new guidelines, which also allow an indication for pharmacotherapy in cases with moderate ADHD, reached 97.2% of criteria fulfilment (Table 2). No differences between professional groups were found concerning TI_OLD_. For TI_NEW_, the estimated marginal mean (EMM) for GA of paediatricians lay at 92.5%, which was significantly lower than that of psychotherapists (EMM = 100%, *β* = 7.48, SE = 3.66, *p* = 0.044), whereas neither of these groups differed significantly from psychiatrists in this regard (96.8%; Table 3). For the total sample, the EMM for GA was significantly higher with the new guidelines (96.2%) than with the old guidelines (EMM = 85.5%, *β* = − 10.78, SE = 1.22, *p* ≤ 0.001).

The mean percentage of adhering the mandatory recommendations of psychoeducation (i.e. information about ADHD for parents and patients) was 38.9%; the mean for all components was 21.2% (Table 2). Information about ADHD was provided to parents in 42.5% of cases and to patients in 31.1% (Online Resource 3, Table S3). At least one of the two mandatory components was documented in 52.1% of cases. Documentation of optional components of PE lay between no cases (exploring subjective health beliefs with others) and 64.7% (imparting general education strategies to parents). The only significant difference between the professional groups was found between paediatricians (EMM = 14.6%) and psychotherapists (EMM = 26.6%), with the latter achieving higher GA (*β* = 12.00, SE = 3.86, *p* = 0.003).

Pharmacotherapy (PH) was initiated by 30 HCPs for a total of 45 patients. The index consisting of the mandatory components of pharmacotherapy [i.e. selection of preparation, dosage, somatic parameters of patient (height, weight and blood pressure/pulse), information about drug/effects as well as possible side effects provided to parents and patient and information about mode of application provided to parents] averaged 72.7% in all cases treated by the HCPs themselves, and the mean for all components was in 51.0% (Table 2). Fulfilment rates of the ten mandatory components ranged from 97.8% for selection of preparation to 22.2% for informing parents in detail about the mode of application (Online Resource 3, Table S4). At least one of the mandatory components was documented for all self-treated cases (100.0%). Optional components ranged from 8.9% (use of rating scales to document titration process) to 77.8% (minimum of two office visits during titration). On average, five office visits were documented during titration trials, with a maximum of 20 visits. No significant differences were found between paediatricians and psychiatrists (Table 3).

Psychotherapeutic interventions (PT) were carried out by 25 HCPs for a total of 39 patients. The index consisting of the mandatory components of psychotherapy (i.e. parent management training including behaviour analysis, facilitating positive child-parent relationship, formulating effective requests, and alternatively one of the following: applying natural consequences, token economy, or timeout) averaged 43.6% for all self-treated cases, and the mean for all components was 37.9% (Table 2). A behaviour analysis was elaborated on with parents in 15.4% of cases, while the child-parent relationship was discussed in 23.1% of cases and effective requests were documented in 59.0% (Online Resource 3, Table S5). One out of the three alternative components of the mandatory standard was documented for 66.7% cases. 97.4% of the protocols contained information about the enforcement of at least one of the four mandatory components. Optional components ranged from 0% (neuropsychological training with patient) to 79.5% (use of specific therapeutic manuals). For 15.4% of all patients, teachers were included in PT.

### Comparison of globally reported GA by HCPs vs. documented GA in specific cases

The correlations of GA scores based on the global interview (Online Resource 3, Table S10), which describe general GA reported by HCPs, with the GA indices derived from HCPs’ documentation for specific cases reported in this paper (Table 2), were low. Only for assessment (AS) was a statistically significant correlation found for the mandatory standard (*r* = 0.42, *p* ≤ 0.001) and for all GA components (*r* = 0.52, *p* ≤ 0.001).

## Discussion

To our knowledge, the current study is one of only a small number of analyses of GA in routine care of juvenile ADHD, which considers individual, patient-based information as well as global self-reports of HCPs in such detail. Furthermore, we are aware of only one other research group that also analysed reports of HCPs based on individual patients [19, 22, 30]. However, the latter analyses were based on retrospective chart reviews, while the present analysis employed an observational approach with systematic documentations conducted during the course of the assessment and treatment of the patients.

Based on the mandatory guideline components, the GA rates observed and documented in specific cases were moderate for assessment (51.5%), psychoeducation (38.9%) as well as for psychotherapy (43.6%) and pharmacotherapy (72.7%). For treatment indication, rates of GA were good (86.4% based on old guidelines; 97.2% based on new guidelines). The comparison of the GA between the professional groups generally yielded only small differences.

The rate of HCPs who documented adherence with all mandatory components per section for more than half of their patients was as follows: 0% for assessment, 19.2% for psychoeducation, 10.0% for pharmacotherapy and 4.0% for psychotherapy. These findings, based on observed and documented specific cases treated, were much lower than those of globally reported GA by the same HCPs found in the first phase of the study: 46.6% for assessment, 65.8% for psychoeducation, 70.0% for pharmacotherapy and 56.0% for psychotherapy. Several reasons may have contributed to these differences.

Most of the previous studies relied solely on global self-reports of HCPs, and thus struggled with the likelihood of positive response bias or overestimation of actual practice. Therefore, we additionally focused on the documentation of individual care processes and employed the interview in advance as an estimation of this expected method effect. In the second study phase, we endeavoured to collect data that were as unbiased as possible, which is why open-formatted questions dominated the documentation. Of course, one could likewise assume that the protocol itself produced a bias, as HCPs were asked about their practices in specific cases, selected by themselves, and were well aware of the study objectives. This may have led the HCPs to provide (or at least document) more optimal care than they would have given under everyday conditions. However, considering the achieved GA rates in phase 2, the presence of under-documentation may be more likely. With the exception of the only significant correlation between the two strategies, found for assessment (Online Resource 3, Table S10), all other sections seem to confirm the method-dependent effects of over- vs. underestimation. Furthermore, when scrutinising our findings in detail, the presence of a negative documentation bias on certain guideline components seems even more obvious. Therefore, in addition to the previously mentioned rates per section, we also calculated the rate of patients for whom any of several specific components had been documented (e.g. any of the six components regarding the clinical interview). By definition, these rates were much higher than the rates for the individual components (Online Resource 3, Tables S1–S5).

Concerning assessment, the range of adherence to specific guideline components is very large. In this section, for example, HCPs were asked to document the contents from the clinical interview with parents in an open format. This may explain why, for instance, the clarification of ADHD symptoms with parents was only documented in 64.7% of cases. Compared to Epstein et al. [19], we found higher GA for the use of ADHD rating scales during assessment, especially for parents (56.7% vs. 80.8%) but also for additional caregivers (i.e. teachers; 55.5% vs. 60.5%). Epstein et al. [19] reviewed patient charts to verify the use of any rating scales (yes/no), whereas in our study, GA required the active reporting of specific diagnostic instruments which were recommended in the guidelines. Especially regarding parent ratings, our results now rather resemble those of Chan et al. [18], even though their data are based on global information.

For treatment indication, adherence based on the old guidelines was already high (86.4%) and increased to 97.2% based on the new guidelines. Different interpretations of this finding appear to be possible: keeping in mind that data collection for the present study had already been completed prior to the publication of the new guidelines, this finding may indicate the existence of a fruitful interaction between routine care and guideline development. However, for the same reason, these results could reflect the presence of pharmacological overtreatment based on old guidelines—even if justified in individual cases. Another explanation might be that the participating HCPs consider international guidelines in their work as well. Especially with regard to the treatment indication for pharmacotherapy, the new German guidelines are now closer to the UK and US guidelines. However, in 95.8% of cases, HCPs considered psychoeducational treatment as a primary intervention, and psychotherapeutic interventions were indicated in 47.3% of cases. These rates are noticeably higher compared to the findings of Epstein et al. [19], who applied US guidelines in their analyses. In addition to the general conclusion that this finding may reflect differences between health care systems, it could also mean that German HCPs include international guidelines for selected treatment sections.

With regard to psychoeducation, only in 42.5% of cases did documentations explicitly mention educating parents about ADHD symptoms. In this section, a fairly common answer was “parent training”, which did not allow a clear assignment to the specific and content-oriented guideline components for psychoeducation. The general trend of lower rates for psychoeducation of additional caregivers (i.e. teachers) corresponds to the findings of the GEK study, [23] as well as results from the global HCP responses within the first phase of our study [31].

Concerning pharmacotherapy, the finding that somatic parameters (i.e. height, weight, blood pressure) were assessed in only 57.8–68.9% of cases prior to prescribing medication was surprising, particularly as this question was explicitly asked in the protocol. The rates for the use of rating scales during titration trials echo the results of Epstein and co-workers [19]: in 10.8% of cases, parent rating scales were used within the first year of pharmacotherapy, while in our sample, ADHD rating scales were used during titration in 17.8% of cases. In both studies, teacher rating scales were rarely used, with 7.5% [19] and 13.3%, respectively. The low rates for clarifying the use of the prescribed drug in detail (22.2%) and target symptoms (20.0%) with parents may again reflect unspecific answers in the open documentation format (e.g. “medication” was assigned to the component “information about drug/effects”).

For psychotherapy, fulfilment rates for any-conditions of parent management training and patient-based interventions, as well as the “at least one mandatory component” condition were again considerably higher than each single mandatory component. At first glance, the high rate of patient-based interventions may seem surprising, as they are optional according to the ADHD guidelines. However, there might be a confounding effect due to the psychotherapy regulations of the German health insurance companies, which define patient-based interventions as mandatory. The use of treatment manuals was reported quite often, indicating that treatments may have been conducted according to guidelines.

### Limitations

Besides the aforementioned problem of underreporting, further limitations of the present study should be noted. Due to the low participation rate in the documentation phase, the generalisability of our results may be questionable. However, we did not find any differences between the participating and non-participating HCPs during the observational phase with regard to GA scores based on global reports within the previous adherence interview. Nevertheless, as HCPs participated on a voluntary basis, we cannot rule out a potential bias, as we have no information on non-participating HCPs. Moreover, compared to the *n* = 290 HCPs who took part only in the first study phase, the *n* = 73 HCPs who completed both phases were willing to undertake greater effort by documenting processes for each included patient. Furthermore, the latter showed a higher rate of participation in additional ADHD contracts, which suggests that this group had already studied the ADHD guidelines intensively before participating in the study. In addition, these HCPs apparently came to the conclusion that the inclusion of guidelines in ADHD care is a desirable and worthwhile goal. Taken together, selectivity is likely. Therefore, it might be too optimistic to interpret our findings as national practice patterns concerning adherence to guidelines in the routine care of ADHD. However, as Epstein and co-workers [30] already concluded: all these limitations are well known for this type of research and still “must be weighed against the value of conducting such studies”.

## Conclusions

The results of the present study suggest many areas for improvement of GA within the routine care of juvenile ADHD in Germany. Targets for enhancement of GA may be the involvement of teachers and schools in the treatment process, the implementation of psychoeducational methods in general, as well as a careful examination of patients, including monitoring of treatment effects during titration trials. Concerning psychoeducation with teachers, information about ADHD and possible treatment options as well as ADHD-specific behaviour modification strategies in the classroom seem highly desirable. This could be realized, for example, through booklets for teachers, school-based training of teachers in psychoeducational seminars or the integration of teacher-based interventions in a comprehensive multimodal treatment approach (e.g., [14, 32, 33]). Regardless of these findings, additional research is needed to determine the most effective and efficient ways of monitoring treatment processes. Recently, for example, Oxley et al. [34] evaluated a web-based application for monitoring at least physical health parameters. Finally, the relation between GA and clinical outcome remains unclear and needs to be investigated, since the implicit assumption that improved GA results in better treatment outcome is yet to be verified.

## Electronic supplementary material

Below is the link to the electronic supplementary material.

Supplementary material 1 (PDF 352 kb)

Supplementary material 2 (PDF 522 kb)

Supplementary material 3 (PDF 284 kb)
