# Supplementary material for: Guideline adherence in German routine care of children and adolescents with ADHD: an observational study
Source: Eur Child Adolesc Psychiatry. 2020 May 28;30(5):757–68. doi: 10.1007/s00787-020-01559-8 (PMC8060198; doi:10.1007/s00787-020-01559-8)
Supplement: Supplementary file 1 — Supplementary material 1 (PDF 352 kb) [file 787_2020_1559_MOESM1_ESM.pdf]

Supplementary material 1

Protocol for HCPs (German version)

Bitte nicht ausfüllen (projektintern)

ID: ____________________

Zurück am: ________________

Forschungsprojekt ADHS-ImLeiV

Implementierung **lei**tlinienorientierter **V**ersorgung
bei Kindern und Jugendlichen mit ADHS

Dokumentation für Versorger

Heutiges Datum: ______________________________________

Ausgefüllt von: ______________________________________

Name und Vorname des Patienten: ______________________

Geburtsdatum des Patienten: ___________________________

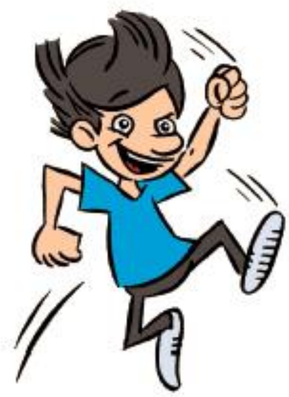

Sehr geehrter Versorger,

in der vorliegenden Dokumentationsvorlage möchten wir sowohl Ihre klinische Einschätzung dieses Patienten als auch die bisher von Ihnen durchgeführten Versorgungsschritte erfassen. Hierbei variiert das Antwortformat zwischen Ankreuzen und Freitext. Zur Vereinfachung wird durchgehend das Maskulinum verwendet, gemeint ist hiermit sowohl die weibliche als auch männliche Form.

Auf den folgenden Seiten werden Sie gebeten, Angaben zu den folgenden drei Bereichen zu machen:

1. **Zuweisungsweg und Vorinformationen**
2. **diagnostisches Vorgehen**
3. **Behandlungsschritte**

Bitte lesen Sie jede Frage sorgfältig durch. Bei den Freitextantworten beschreiben Sie bitte kleinschrittig, wie Sie bei der Versorgung dieses Patienten vorgegangen sind. Dabei genügen jedoch Stichworte! Sollten Sie einzelne Schritte nicht selbst durchgeführt, sondern dafür an Dritte verwiesen haben, machen Sie hierzu die entsprechenden Angaben in den dafür vorgesehenen Feldern.

Um Ihnen unnötige Fragen zu ersparen, haben wir an machen Stellen Hinweise zum Überspringen von Abschnitten eingefügt. Überspringen Sie daher bitte nie ganze Themenbereiche, sondern beginnen Sie **immer** mit der Beantwortung der ersten Fragen, bis Sie auf einen solchen Verweis stoßen. Ab hier folgen Sie dann bitte dem für Sie angemessen Weg!

Falls Sie Schwierigkeiten bei der Bearbeitung haben, stehen wir Ihnen gerne unter 0221 478 87774 für Rückfragen zur Verfügung.

Wir möchten Sie an dieser Stelle erneut darauf hinweisen, dass Ihre Angaben selbstverständlich im Rahmen der späteren gruppenstatistischen Auswertung **pseudonymisiert** und somit nicht mehr zu identifizieren sein werden.

Und nun zu den Fragen…

ZUWEISUNGSWEG UND AUSGANGSINFORMATIONEN			
I	Wie kam der Patient zu Ihnen?<br>☐1 Erstkonsultation ohne Überweisung wegen (V.a.) ADHS<br>☐2 Erstkonsultation ohne Überweisung wegen anderer Beschwerden als (V.a.) ADHS<br>☐3 Erstkonsultation mit Überweisung wegen (V.a.) ADHS<br>☐4 Erstkonsultation mit Überweisung wegen anderer Beschwerden als (V.a.) ADHS<br>☐5 Bereits aus anderen Gründen Patient in der Praxis/Einrichtung		
I.a	Bei Überweisung bitte beschreiben (z.B. von Kinder- & Jugendmediziner):		
I.b	☐6 Sonstiges (bitte beschreiben):		
II	Gesicherte Diagnose einer ADHS lag zum Zeitpunkt der Erstvorstellung bereits vor:	☐0 Nein	☐1 Ja
III	Ausführlicher Befundbericht von dritter Seite liegt vor:	☐0 Nein	☐1 Ja
IV	Befunde zu standardisierten Testverfahren liegen vor:	☐0 Nein (zu V)	☐1 Ja (zu IV.a)
IV.a	Bitte beschreiben:		
V	Wenn bereits eine gesicherte Diagnose vorlag, sahen Sie die Notwendigkeit diese zu überprüfen?	☐0 Nein (zu VI)	☐1 Ja (zu V.a)
V.a	Bitte begründen:		
VI	Befand sich der Patient zum Zeitpunkt der Vorstellung wegen ADHS bereits an anderer Stelle in Diagnostik/Behandlung?	☐0 Nein (zu VII)	☐1 Ja (zu VI.a-g)
VI.a	☐1 Pharmakotherapie[1)]		
VI.b/c	Wenn bekannt, Medikament:	Tagesdosis:	mg
VI.d	☐1 Psychoedukation & Beratung[2)]		
VI.e/f	☐1 Psychotherapie[3)]<br>Wenn bekannt, therapeutische Ausrichtung:		
VI.g	☐1 Diagnostische Abklärung		
VI.h	☐1 Sonstiges[4)] (Bitte beschreiben):		

[1)] Rezeptpflichtige Neuroleptika (z.B. Stimulanzien, Amphetamine, Atomoxetin, Neuroleptika); [2)]durch qualifiziertes Fachpersonal, sofern diese mit Krankenkassen abgerechnet werden (z.B. Elterntrainings); [3)]Richtlinienpsychotherapie durch approbierte Psychotherapeuten oder Therapeuten in Ausbildung, Fachärzte für Kinder-und Jugendpsychiatrie & Psychotherapie oder Ärzte mit Zusatzqualifikation „Psychotherapie“; schließt Psychoedukation & Beratung ein; [4)]Übungsbehandlungen (z.B. Ergotherapie, Konzentrationstraining), alternative Ansätze (z.B. diätetische Maßnahmen)

VERSORGUNG BEI IHNEN						
VII						
1. Termin am:	___________	Dauer (in Min.)	________	☐ Patient	☐ Eltern	☐ Andere
2. Termin am:	___________	Dauer (in Min.)	________	☐ Patient	☐ Eltern	☐ Andere
3. Termin am:	___________	Dauer (in Min.)	________	☐ Patient	☐ Eltern	☐ Andere
4. Termin am:	___________	Dauer (in Min.)	________	☐ Patient	☐ Eltern	☐ Andere
5. Termin am:	___________	Dauer (in Min.)	________	☐ Patient	☐ Eltern	☐ Andere
6. Termin am:	___________	Dauer (in Min.)	________	☐ Patient	☐ Eltern	☐ Andere
7. Termin am:	___________	Dauer (in Min.)	________	☐ Patient	☐ Eltern	☐ Andere
8. Termin am:	___________	Dauer (in Min.)	________	☐ Patient	☐ Eltern	☐ Andere
9. Termin am:	___________	Dauer (in Min.)	________	☐ Patient	☐ Eltern	☐ Andere
10. Termin am:	___________	Dauer (in Min.)	________	☐ Patient	☐ Eltern	☐ Andere
11. Termin am:	___________	Dauer (in Min.)	________	☐ Patient	☐ Eltern	☐ Andere
12. Termin am:	___________	Dauer (in Min.)	________	☐ Patient	☐ Eltern	☐ Andere
13. Termin am:	___________	Dauer (in Min.)	________	☐ Patient	☐ Eltern	☐ Andere
14. Termin am:	___________	Dauer (in Min.)	________	☐ Patient	☐ Eltern	☐ Andere
15. Termin am:	___________	Dauer (in Min.)	________	☐ Patient	☐ Eltern	☐ Andere
16. Termin am:	___________	Dauer (in Min.)	________	☐ Patient	☐ Eltern	☐ Andere
17. Termin am:	___________	Dauer (in Min.)	________	☐ Patient	☐ Eltern	☐ Andere
18. Termin am:	___________	Dauer (in Min.)	________	☐ Patient	☐ Eltern	☐ Andere
19. Termin am:	___________	Dauer (in Min.)	________	☐ Patient	☐ Eltern	☐ Andere
20. Termin am:	___________	Dauer (in Min.)	________	☐ Patient	☐ Eltern	☐ Andere
21. Termin am:	___________	Dauer (in Min.)	________	☐ Patient	☐ Eltern	☐ Andere
22. Termin am:	___________	Dauer (in Min.)	________	☐ Patient	☐ Eltern	☐ Andere
23. Termin am:	___________	Dauer (in Min.)	________	☐ Patient	☐ Eltern	☐ Andere
24. Termin am:	___________	Dauer (in Min.)	________	☐ Patient	☐ Eltern	☐ Andere

VIII	Wird es weitere Termine bzgl. der ADHS- Symptomatik geben? ☐0 Nein (zu VIII.a-g) ☐1 Ja (nächste Seite)
VIII.a-g	Bitte begründen Sie die Beendigung der Behandlung (Mehrfachnennungen möglich):<br>☐1 regulär beendet<br>☐1 Behandlung nicht wirksam<br>☐1 in alternative Behandlung überwiesen<br>☐1 Umzug/mangelnde Erreichbarkeit der Familie<br>☐1 Abbruch durch mich<br>☐1 Abbruch durch Eltern/Patient<br>☐1 Sonstiges (zu VIII.h)
VIII.h	Bitte beschreiben:

DIAGNOSTISCHE PHASE<br>Angaben zu den vergangenen 3 Monaten		
01	Wurden diagnostische Maßnahmen hinsichtlich der Abklärung von ADHS oder anderer psychischer Verhaltensauffälligkeiten durchgeführt?<br>☐$_{0}$ Nein (weiter zu 08)<br>☐$_{1}$ Ja, dafür habe ich verwiesen (weiter zu 03)<br>☐$_{2}$ Ja, von mir selbst (weiter zu 02)	
02	Wie viel Zeit haben Sie für die Diagnosestellung aufgewendet: ________ Minuten	
03	Wenn Sie zu diagnostischen Zwecken verwiesen haben, erfolgte dies:<br>☐$_{1}$ intern oder ☐$_{2}$ extern	
03a	Bitte geben Sie die Berufsgruppe des Kollegen an: ________________________________	
	Welche Inhalte/Themen wurden im Rahmen von Exploration & Anamnese abgeklärt bzw. waren Ihnen zu diesem Zeitpunkt bekannt?	
	durch Sie	durch Andere (sofern bekannt)
04a/b	mit Eltern:	
04c/d	mit Patienten:	
04e/f	mit Anderen:	
05	Wurden standardisierte Fragebogenverfahren eingesetzt?<br>☐$_{0}$ Nein (weiter zu 06)<br>☐$_{1}$ Ja, dafür habe ich verwiesen (weiter zu 05 b, d, f)<br>☐$_{2}$ Ja, von mir selbst (weiter zu 05 a, c, e)	
	durch Sie	durch Andere (sofern bekannt)
05a/b	mit Eltern:	
05c/d	mit Patienten:	
05e/f	mit Anderen:	

06	Wurden weitere psychologische Testverfahren eingesetzt?<br><br>☐0 Nein (weiter zu 07)<br>☐1 Ja, dafür habe ich verwiesen (weiter zu 06 b, d, f)<br>☐2 Ja, von mir selbst (weiter zu 06 a, c, e)	
	durch Sie	durch Andere (sofern bekannt)
06a/b	mit Eltern:	
06c/d	mit Patienten:	
06e/f	mit Anderen:	
07	Wurden körperliche Untersuchungen am Patienten durchgeführt?<br><br>☐0 Nein (weiter zu 08)<br>☐1 Ja, dafür habe ich verwiesen (weiter zu 07b)<br>☐2 Ja, von mir selbst (weiter zu 07a)	
	durch Sie	durch Andere (sofern bekannt)
07a/b	mit Patienten:	

IHRE PERSÖNLICHE GESAMTEINSCHÄTZUNG

08	Kann/Können eine/mehrere Diagnose(n) psychischer Störung(en) (inkl. Entwicklungsstörungen & Intelligenzminderungen) gestellt werden?	☐0 Nein (zu 09)	☐1 Ja (zu 08a-f)		
08a/b	F ____________	Schweregrad:	☐1 leicht	☐2 mittel	☐3 schwer
08c/d	F ____________	Schweregrad:	☐1 leicht	☐2 mittel	☐3 schwer
08e/f	F ____________	Schweregrad:	☐1 leicht	☐2 mittel	☐3 schwer
09	Gibt es weitere diagnostische Maßnahmen/ Verfahren, die Sie bei diesem Patienten für sinnvoll erachtet hätten?	☐0 Nein (zu 10)	☐1 Ja (zu 09a)		
09a	Nennen Sie uns bitte kurz die betreffenden Maßnahmen und die Gründe warum diese nicht umgesetzt wurden/ werden konnten.<br><br>Bitte beschreiben:				

**INDIKATIONSSTELLUNG**<br>**Angaben zu den vergangenen 3 Monaten**				
	Bei dem Patienten bestand nach Abschluss der Diagnostik eine Indikation für:			
10a	Pharmakotherapie[1)]	☐$_0$ Nein	☐$_1$ primär	☐$_2$ ggf. später
10b	Psychoedukation & Beratung[2)]	☐$_0$ Nein	☐$_1$ primär	☐$_2$ ggf. später
10c	Psychotherapie[3)]	☐$_0$ Nein	☐$_1$ primär	☐$_2$ ggf. später
10d	Sonstiges[4)]<br>(Bitte beschreiben):	☐$_0$ Nein	☐$_1$ primär	☐$_2$ ggf. später

	In den vergangenen 3 Monaten wurde bereits eine Behandlung durch Sie begonnen/eingeleitet	bei Ihnen	bei Anderen (sofern bekannt)			
			intern<br>(eigene Praxis/Einrichtung)		extern<br>(andere Praxis/Einrichtung)	
11a	Pharmakotherapie[1)]	☐$_1$ Ja	☐$_2$ Nein	☐$_3$ Ja	☐$_4$ Nein	☐$_5$ Ja
11b	Psychoedukation & Beratung[2)]	☐$_1$ Ja	☐$_2$ Nein	☐$_3$ Ja	☐$_4$ Nein	☐$_5$ Ja
11c	Psychotherapie[3)]	☐$_1$ Ja	☐$_2$ Nein	☐$_3$ Ja	☐$_4$ Nein	☐$_5$ Ja
11d	Sonstiges[4)]<br>(Bitte beschreiben):	☐$_1$ Ja	☐$_2$ Nein	☐$_3$ Ja	☐$_4$ Nein	☐$_5$ Ja

12	Wurde zunächst ein anderer Behandlungsschritt eingeleitet, als nach Ihrer diagnostischen Entscheidung primär indiziert war?	☐$_0$ Nein (zu 13)	☐$_1$ Ja (zu 12a)
12a	Bitte begründen:		

**PSYCHOEDUKATION UND BERATUNG**		
13	Wurden psychoedukative oder beratende Inhalte/Themen bereits erörtert?<br>☐$_0$ Nein (weiter zu 14)<br>☐$_1$ Ja, dafür habe ich verwiesen. (weiter zu 13 b, d, f)<br>☐$_2$ Ja, von mir selbst. (weiter zu 13 a, c, e)	
	durch Sie	durch Andere (sofern bekannt)
13a/b	mit Eltern:	
13c/d	mit Patienten:	
13e/f	mit Anderen:	

[1)] Rezeptpflichtige Neuroleptika (z.B. Stimulanzien, Amphetamine, Atomoxetin, Neuroleptika); [2)]durch qualifiziertes Fachpersonal, sofern diese mit Krankenkassen abgerechnet werden (z.B. Elterntrainings); [3)]Richtlinienpsychotherapie durch approbierte Psychotherapeuten oder Therapeuten in Ausbildung, Fachärzte für Kinder-und Jugendpsychiatrie & Psychotherapie oder Ärzte mit Zusatzqualifikation „Psychotherapie“; schließt Psychoedukation & Beratung ein; [4)]Übungsbehandlungen (z.B. Ergotherapie, Konzentrationstraining), alternative Ansätze (z.B. diätetische Maßnahmen)

14	Gibt es (weitere) psychoedukative/beratende Inhalte/Themen, die Sie für sinnvoll erachtet hätten?	☐0 Nein (zu 15) ☐1 Ja (zu 14a)
14a	Nennen Sie uns bitte kurz die betreffenden Inhalte/Themen und die Gründe warum diese nicht umgesetzt wurden/ werden konnten.<br>Bitte beschreiben:	
**PHARMAKOTHERAPIE**		
15	Wird der Patient zurzeit medikamentös wegen ADHS behandelt?<br>☐0 Nein (weiter zu 15a)<br>☐1 Ja, dafür habe ich verwiesen (weiter zu 15b).<br>☐2 Ja, von mir selbst (weiter zu 15c).	
15a	Wenn zurzeit keine medikamentöse Behandlung erfolgt, machen Sie hierzu bitte genauere Angaben:<br>☐0 Nein, noch nie – soweit bekannt – (weiter zu 23)<br>☐1 Behandlungsversuch innerhalb der vergangenen 3 Monate beendet (weiter zu 15c)<br>☐2 Aktueller Auslassversuch (weiter zu 15c)	
15b	Wenn Sie verwiesen haben, geben Sie bitte an, ob Ihnen von anderer Stelle Informationen übermittelt wurden:	☐0 Nein (zu 23) ☐1 Ja (zu 15c)
15c	Geben Sie die aktuelle oder letzte Medikation und Dosierung an:<br>Medikament: Tagesdosis: mg<br>Weitere Informationen:	

Die folgenden Fragen müssen Sie nur beantworten, wenn Sie selbst eine Pharmakotherapie beim Patienten

- **bereits begonnen haben,**
- **oder einen Behandlungsversuch beendet haben**
- **oder derzeit ein Auslassversuch durchgeführt wird.**

Falls dies nicht zutrifft, springen Sie bitte zu Frage 23.

16	Bitte geben Sie die Anzahl der Kontakte an (direkt, telefonisch), in denen die Ermittlung der optimalen Dosis für den Patienten Thema war:	_____ Kontakte
17	Wurden spezifische körperliche Parameter überprüft?	☐0 Nein (zu 18) ☐1 Ja (zu 17a)
17a	Welche körperlichen Parameter und zu welchem(n) Zeitpunkt(en):<br>Bitte beschreiben:	
18	Wurden im Rahmen der medikamentösen Behandlung spezifische Inhalte/Themen abgeklärt?	☐0 Nein (zu 20) ☐1 Ja (zu 18 a-c)
18a	mit den Eltern:	
18b	mit den Patienten:	
18c	mit Anderen:	
19	Wurden hierbei standardisierte Fragebögen eingesetzt?	☐0 Nein (zu 20a-c) ☐1 Ja (zu 19a)
19a	Bitte beschreiben:	
	Welche Informationen haben Sie im Rahmen der medikamentösen Behandlung vermittelt?	
20a	an die Eltern:	
20b	an Patienten:	
20c	an Andere:	
21	Bitte beschreiben Sie, wie die derzeitige Tagesdosis ermittelt wurde:	

22	Wurde innerhalb der vergangenen 3 Monate ein Auslassversuch durchgeführt?	☐0 Nein (zu 23)	☐1 Ja (zu 22a)
22a	Bitte beschreiben Sie den Ablauf:		
23	Gibt es (weitere) Maßnahmen/Verfahren, die Sie im Zusammenhang mit der medikamentösen Behandlung für sinnvoll erachtet hätten?	☐0 Nein (zu 24)	☐1 Ja (zu 23 a)
23a	Nennen Sie uns bitte kurz die betreffenden Inhalte/Themen und die Gründe warum diese nicht besprochen wurden/ werden konnten.<br>Bitte beschreiben:		
**PSYCHOTHERAPIE**			
24	Wird der Patient zurzeit bzgl. der ADHS-Symptomatik psychotherapeutisch[5)] behandelt?<br>☐0 Nein<br>☐1 Ja, dafür habe ich verwiesen.<br>☐2 Ja, von mir selbst.		
25	Wird der Patient zurzeit wg. komorbider Symptomatiken psychotherapeutisch behandelt?<br>☐0 Nein<br>☐1 Ja, dafür habe ich verwiesen. (weiter zu 26)<br>☐2 Ja, von mir selbst. (weiter zu 27)		
**Haben Sie die beiden letzten Fragen mit „Nein“ beantwortet?<br>Dann sind Sie fast fertig (weiter zu 30)!**<br><br>**Aber jetzt ist es auch für alle anderen gleich geschafft ☺**			
26	Wenn Sie verwiesen haben, geben Sie bitte an, ob Ihnen Informationen von anderer Stelle übermittelt wurden:	☐0 Nein	☐1 Ja (zu 26a)
26a	Bitte beschreiben:		
**Die folgenden Fragen müssen Sie nur beantworten, wenn Sie selbst eine entsprechende Behandlung bei diesem Patienten bereits begonnen haben.**<br><br>**Falls dies nicht zutrifft, springen Sie bitte zu Frage 30.**			

5) Richtlinienpsychotherapie durch approbierte Psychotherapeuten oder Psychotherapeuten in Ausbildung, Fachärzte für Kinder- und Jugendpsychiatrie und Psychotherapie oder Ärzte mit Zusatzqualifikation „Psychotherapie“; schließt Psychoedukation & Beratung ein

27	Wurden im Rahmen der Psychotherapie spezifische Inhalte/Themen oder Ziele besprochen?	☐0 Nein (zu 28)	☐1 Ja (zu 27a-c)
27a	mit Eltern:		
27b	mit Patienten:		
27c	mit Anderen:		
28	Wurden im Rahmen der Psychotherapie der ADHS-Symptome spezifische Interventionen durchgeführt?	☐0 Nein (zu 29)	☐1 Ja (zu 28a-c)
28a	mit Eltern:		
28b	mit Patienten:		
28c	mit Anderen:		
29	Haben Sie dabei therapeutische Manuale eingesetzt?	☐0 Nein (zu 30)	☐1 Ja (zu 29a)
29a	Bitte beschreiben:		
30	Gibt es (weitere) Maßnahmen/Verfahren, die Sie im Zusammenhang mit der psychotherapeutischen Behandlung für sinnvoll erachtet hätten?	☐0 Nein	☐1 Ja (zu 30a)
30a	Nennen Sie uns bitte kurz die betreffenden Inhalte/Themen und die Gründe warum diese nicht besprochen wurden/ werden konnten.<br>Bitte beschreiben:		

**Vielen Dank, dass Sie sich die Zeit genommen haben,
diese Dokumentationsvorlage auszufüllen!**
