# Supplementary material for: Guideline adherence in German routine care of children and adolescents with ADHD: an observational study
Source: Eur Child Adolesc Psychiatry. 2020 May 28;30(5):757–68. doi: 10.1007/s00787-020-01559-8 (PMC8060198; doi:10.1007/s00787-020-01559-8)
Supplement: Supplementary file 2 — Supplementary material 2 (PDF 522 kb) [file 787_2020_1559_MOESM2_ESM.pdf]

Supplementary material 2

Rating manual (German version)

Bitte nicht ausfüllen (projektintern)

ID: ____________________

Zurück am: ________________

Forschungsprojekt ADHS-ImLeiV

Implementierung **lei**tlinienorientierter **V**ersorgung

bei Kindern und Jugendlichen mit ADHS

Rating-Manual

(Bewertungsleitfaden Versorger-Dokumentation)

Heutiges Datum: ____

Ausgefüllt von: ____

Name und Vorname des Patienten: ______________________

Geburtsdatum des Patienten: ___________________________

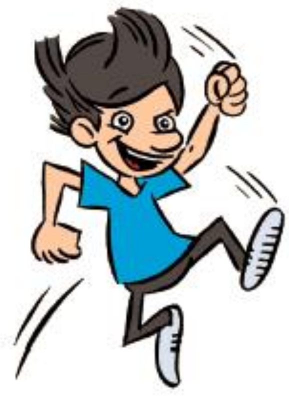

GRUNDREGELN FÜR DIE EINGABE IN SPSS:

1.1 ALLGEMEINES

Wann steht eine Angabe an der „falschen“ Stelle?

Immer dann, wenn eine Angabe in einem Themenbereich genannt wird, in der es gemäß der Vorgaben keine Punkte geben würde, in einem anderen Themenbereich aber sehr wohl kodierbar wäre.

Wie geht man vor, wenn eine Angabe an „falscher“ Stelle steht?

Grundsätzlich sollten Punkte „gerettet“ werden, und dorthin verschoben werden, wo es Punkte für diese Angabe geben würde. Trotzdem sollte nur dann korrigiert/verschoben werden, wenn die Zuweisung zum korrekten Item **zweifelsfrei** möglich ist. Wenn ein Punkt verschoben wird, wird dies an beiden Stellen („Falsche“ und Verschobene) im Dokumentationsbogen vermerkt!

Wie geht man mit Unstimmigkeiten bei der Frage um, durch wen die jeweilige Intervention erfolgte?

Sollte ersichtlich sein, dass bestimmte Behandlungsschritte sowohl vom Versorger selbst, als auch von jemand anderem durchgeführt worden sind, bei der entsprechenden Frage dazu aber nur eine von beiden Angaben gemacht wurde, wird diese immer zu „3 = ja von mir selbst und verwiesen (1 und 2)“ korrigiert.

Wie geht man mit Dopplungen bei der Frage um, durch wen die jeweilige Intervention erfolgte?

Sollten sich bei einer Frage die Angaben des Versorgers bei „durch Sie“ und „durch Andere“ doppeln, wird dies auch genauso eingegeben. Diese Dopplungen stellen kein Problem dar, da die Syntax das später automatisch prüfen wird.

Wie wird mit Angaben im Themenbereich „Zuweisungsweg und Ausgangsinformationen“ umgegangen?

Sofern Angaben zu Vorbefunden (z.B. Fragebögen oder Leistungsdiagnostik) gemacht worden sind, werden diese zu den entsprechenden Items unter (zumeist) Diagnostik übertragen und dort bepunktet.

Wie gehe ich mit Angaben bei „mit Anderen“ um, wenn klar ist, dass es sich hier um Pflege- oder Großeltern handelt?

In diesem Fall werden die Angaben in sämtlichen Kapiteln zu „mit Eltern“ verschoben und dort bepunktet, da hiermit stets die ersten Bezugspersonen für das Kind gemeint sind. Andernfalls würde ein ungerechtfertigter Punkteverlust resultieren, da man unter „mit Anderen“ meistens nur einen Standardpunkt bekommt, sofern überhaupt mit diesen Kontakt aufgenommen wurde (s. auch Terminangaben).

Was ist, wenn die Angaben im Fragebogen abrupt enden?

In diesem Fall wird von einem händischen ergänzen durch die Rater abgesehen; an notwendigen Stellen werden Missings später durch die Syntax ersetzt werden.

Was ist, wenn sich Angaben aus M3 in M6 wiederholen?

Derartige Angaben können sowohl in M3 als auch in M6 kodiert werden. Wiederholte bzw. ähnliche Angaben wird später die Syntax überprüfen, damit dann nur ein Punkt vergeben wird.

Was ist, wenn im ADHS-KGE (Fragebogenteil der M6) bei „weitere“ Formulierungen auftauchen, die kodierbar wären?

Konkretes Beispiel: Angabe, dass sich Eigensteuerung und Selbstständigkeit verbessert haben. Eine Kodierung dieser Angabe bei AUQ oder PT findet **nicht** statt, sofern der Versorger nicht in den entsprechenden Kapiteln eindeutig angegeben hat, dass er durch bestimmte Interventionen **aktiv** an dieser Besserung beteiligt war.

1.2 TERMINANGABEN

Was ist wenn der Versorger 2 Termine am selben Tag angibt?

Wenn der Versorger dies explizit benennt, werden diese auch getrennt und doppelt eingetragen. Wenn der Versorger dies nicht tut, beispielsweise eine Termindauer von 200 Minuten angibt, dann wird es so übernommen, wie es da steht.

Was ist, wenn der Versorger zu M6 noch einmal sämtliche stattgefundenen Termine benennt?

Zu M6 werden ausschließlich jene Termine eingetragen, die nach M3 stattgefunden haben. Die vorherigen Termine werden nicht doppelt erfasst, da die ermittelte Summe zur Terminanzahl sonst zu hoch ausfallen würde.

Was ist wenn sich die Terminangaben in M3 und M6 widersprechen?

Sollte der Versorger in der M6 noch einmal sämtliche Termine über den vollständigen 6-monatigen Erhebungszeitraum angegeben haben und sich diese dann im Abgleich mit M3 in Angaben der Dauer, der Anwesenden oder sogar im Datum unterscheiden, so werden die Termine, so wie sie in M3 dokumentiert wurden in der M3 Datenmaske beibehalten. In die M6 Datenmaske werden nur die Termine ab M6 eingegeben (s.o.)

Was ist, wenn der Versorger nur zu M6 Termine benennt, da keine (vollständige) M3 vorliegt?

In diesem Fall werden die Termine, die *vor* der Aussendung von M3 stattgefunden haben, auch in der M3 Datenmaske eingegeben. Alle übrigen Angaben werden in der M6 Datei kodiert, da nicht mehr nachvollziehbar ist, welche Maßnahme zu welchem Zeitpunkt stattgefunden hat. Bei der Verbindung beider Dateien wird dies aber ohnehin obsolet.

Was ist, wenn nach dem ersten Termin nur noch die Angabe „wöchentliche Termine“ dokumentiert ist?

Wenn der Versorger angibt, von bspw. 18.03.15 an wöchentliche Termine á 30 Minuten mit dem Patienten gehabt zu haben, dann werden diese Angaben unter Zuhilfenahme eines Kalenders bis zum Ausfülldatum der betreffenden Dokumentation ergänzt und in SPSS eingetragen. Sofern möglich sollten Feiertage und Ferien ausgelassen werden.

Wie gehe ich mit der Angabe „Andere“ um, wenn es sich hierbei nachweislich um Pflegeeltern handelt?

In diesem Fall wird die Angabe korrigiert und in SPSS bei Anwesende als „Eltern“ verbucht (s. auch Allgemeines)

Wie gehe ich mit Angaben wie „Elterntraining“, „Neurofeedback“, „Gruppentherapie“ um?

Sofern diese Termine nachweislich in der eigenen Praxis/Einrichtung des Versorgers stattgefunden haben, werden diese komplett eingegeben, sofern konkrete Terminangaben gemacht worden sind. Wenn dies aus den Angaben nicht eindeutig hervorgeht oder die Termine zweifelsfrei nicht in der eigenen Praxis stattgefunden haben muss die Eingabe in SPSS entfallen.

1.3 BARRIEREN

<u>Wann wird welcher Barrieren-Grund verschlüsselt?</u>

Sämtliche Möglichkeiten zur Verschlüsselung sind aus Sicht des Versorgers zu verstehen und sollten entsprechend verschlüsselt werden.

Beispiel: Der Versorger gibt an, dass eine Ergotherapie aus Zeitmangel eines **externen** Kollegen noch nicht begonnen hat, dann wird diese Barriere **nicht** unter Zeitmangel verschlüsselt, da es sich nicht um eine Zeitnot der teilnehmenden EinricH-tung/Praxis handelt. Stattdessen würde in diesem Fall „Kooperationsmangel“ verschlüsselt werden. Wenn es sich um einen **internen** Kollegen handelt wird hingegen „Zeitmangel“ kodiert.

Zeitmangel *wird immer dann verschlüsselt, wenn:*

1. Sich Angaben finden wie „Diagnostik noch nicht abgeschlossen“ oder „PT kann erst am XX.XX.XXXX beginnen“. Voraussetzung hierfür ist wie oben beschrieben, dass die jeweiligen Interventionen intern stattfinden sollen. Bei derartigen Formulierungen die sich auf extern durchgeführte Interventionen beziehen → s. Koopmangel
2. Die Formulierung „steht auf Warteliste“ zu finden ist, sofern es sich um eine interne Warteliste handelt. Sollte sich die Angabe auf eine Warteliste bei einem externen Kollegen beziehen → s. Koopmangel

Mangel an Kooperationsmöglichkeiten *wird immer dann verschlüsselt, wenn:*

1. Es aktuell keine freien Plätze bei Kollegen in der Umgebung gibt
2. Der Kollege bislang nicht mit der Behandlung begonnen hat bzw. der Patient dort auf der Warteliste steht
3. Der Versorger mit den bisher durchgeführten Interventionen des Kollegen nicht einverstanden ist

Familienbezogene Gründe *werden immer dann verschlüsselt, wenn:*

1. Irgendein Behandlungsschritt wegen mangelnder Compliance oder aus zeitlichen Gründen des Patienten nicht oder nicht zufriedenstellend durchgeführt wurde
2. Irgendein Behandlungsschritt wegen mangelnder Compliance oder aus zeitlichen Gründen der Bezugspersonen nicht oder nicht zufriedenstellend durchgeführt wurde

Organisatorische Gründe *werden immer dann verschlüsselt, wenn:*

1. Aus der Angabe eindeutig ersichtlich wird, dass eine bestimmte Intervention niemals in der jeweiligen Einrichtung/Praxis durchgeführt wird, beispielsweise weil die Befugnis fehlt. Es sei denn diese Tatsache wird auf Kenntnismangel zurück geführt, dann wird die Angabe dort verschlüsselt.

<u>Was ist, wenn eine Angabe keiner eindeutigen Verschlüsselungsoption zugewiesen werden kann?</u>

In diesem Fall gibt es mehrere Dinge zu überprüfen:

1. Handelt es sich tatsächlich um die Angabe einer Barriere?

Es kann sein, dass diese Felder für allgemeine Anmerkungen genutzt wurden.
Nur in vollkommen eindeutigen Fällen, wird die Angabe zwar in der String-Variable eingetragen, aber **nicht** gerated. Zudem wird bei dem vorgeschalteten Barriere Ja/Nein-Item eine „0“ kodiert.

2. Es handelt sich um eine Barriere aber diese ist nicht ausreichend begründet?

In manchen Fällen finden sich unter den Barrieren ungenaue Angaben.

Beispiel: Eine Formulierung wie „systemische Familientherapie". In diesem Fall ist es nicht möglich zu entscheiden, wie diese Angaben verschlüsselt werden soll. Dann wird der Freitext nicht in die Stringvariable _1b sondern in der zweiten Stringvariable _1d unter „Sonstige Barrieren" eingetragen. Das vorgeschaltete Ja/Nein-Item erhält in diesem Fall eine „1".

Was ist, wenn sich durch den Abgleich von M3 und M6 eine mögliche Verschlüsselungsoption ergeben könnte?

Sollte dieser Fall auftreten, wird er ignoriert! Es findet also keine Übertragung von Barriere-Gründen statt. In der Doku, in der die Angabe uneindeutig ist, wird diese unter „Sonstige" eingetra gen (s.o.) und in der eindeutigen Doku wie gewohnt kodiert.

Können für dieselbe Angabe mehrere Barrieren-Gründe verschlüsselt werden?

Grundsätzlich sollte hiervon abgesehen werden, beispielsweise wenn es sich nur um einen Satz handelt, sollte dieser eindeutig verschlüsselt werden. Nur in wenigen Ausnahmefällen und einer sehr ausführlichen Schilderung, die eindeutig mehrere Gründe beinhaltet, ist eine Doppelkodierung denkbar.

Wann steht eine Barriere an „falscher" Stelle?

Sofern es sich um recht allgemeine Angaben zu Behandlungsschritten, die beispielweise aus Patienten- oder Kooperationsgründen nicht eingeleitet werden konnten handelt, gehören diese **immer** zum Bereich „Barrieren Indikation". Sollten spezifischere Interventionsschritte benannt sein, beispielsweise Probleme bei der Eindosierung oder beim Einführen eines Punkte-Plans gehören diese **immer** in die entsprechenden Kapitel „ Barrieren Pharmako" bzw. „Barrieren Psychotherapie".

Wie geht man vor, wenn eine Barriere an „falscher" Stelle steht?

Angegebene Barrieren sollten durch den Rater verschoben werden, sofern sie eindeutig an eine andere Stelle gehören. Bei Uneindeutigkeit schlägt der Freitext sämtliche Kreuzchen bei den Ja/Nein-Items sowie den Angaben unter Indikation, sofern dieser eine eindeutige Erklärung bietet.

Beispiel: Bei Barrieren Pharmako wird erwähnt, dass die Eltern Medikamente abgelehnt haben. In diesem Fall wird die Angabe zu Indikation verschoben und mit „Patientengründe" verschlüsselt. Diese Änderung wird sowohl in der Dokumentation als auch in SPSS mit „VON PHB" und „NACH INB" festgehalten.

In einigen Fällen könnten sich hierdurch fehlende Angaben im Bereich der Indikation ergeben. Sofern diese eindeutig identifizierbar sind, soll hier korrigiert werden.

Beispiel: Der Versorger gibt an, aus mangelnder Compliance keine Ergotherapie eingeleitet haben zu können, die Indikationsangabe für Ergotherapie fehlt aber unter „Sonstige" → dann ergänzen!

Weitere Rating-Regeln finden sich den jeweiligen Items direkt zugeordnet und sind mit einem grünen Kasten gekennzeichnet!

ZUWEISUNGSWEG UND AUSGANGSINFORMATIONEN			
I	Wie kam der Patient zu Ihnen?<br>☐1 Erstkonsultation ohne Überweisung wegen (V.a.) ADHS<br>☐2 Erstkonsultation ohne Überweisung wegen anderer Beschwerden als (V.a.) ADHS<br>☐3 Erstkonsultation mit Überweisung wegen (V.a.) ADHS<br>☐4 Erstkonsultation mit Überweisung wegen anderer Beschwerden als (V.a.) ADHS<br>☐5 Bereits aus anderen Gründen Patient in der Praxis/Einrichtung		
I.a	Bei Überweisung bitte beschreiben (z.B. von Kinder- und Jugendmediziner)<br>**Wertelabels:**<br>**1 SPZ**<br>**2 Klinikambulanz**<br>**3 KJ-Medizinische Praxis**<br>**4 KJ-Psychiatrische Praxis**<br>**5 KJ-Psychotherapie Praxis**<br>**6 Ausbildungsambulanz**<br>**7 Sonstiges**		
I.b	☐6 Sonstiges (bitte beschreiben): **Freitext**		
II	Gesicherte Diagnose einer ADHS lag zum Zeitpunkt der Erstvorstellung bereits vor:	☐0 **Nein**	☐1 **Ja**
III	Ausführlicher Befundbericht von dritter Seite liegt vor:	☐0 **Nein**	☐1 **Ja**
IV	Befunde zu standardisierten Testverfahren liegen vor:	☐0 **Nein** (zu V)	☐1 **Ja** (zu IV.a)
IV.a	Bitte beschreiben: **Freitext**		
V	Wenn bereits eine gesicherte Diagnose vorlag, sahen Sie die Notwendigkeit diese zu überprüfen?	☐0 **Nein** (zu VI)	☐1 **Ja** (zu V.a)
V.a	Bitte begründen: **Freitext**		
VI	Befand sich der Patient zum Zeitpunkt der Vorstellung wegen ADHS bereits an anderer Stelle in Diagnostik/Behandlung?	☐0 **Nein** (zu VII)	☐1 **Ja** (zu VI.a-g)
VI.a	Pharmakotherapie	☐0 **Nein**	☐1 **Ja**
VI.b/c	Wenn bekannt, Medikament1: Tagesdosis: mg<br>Wenn bekannt, Medikament2: Tagesdosis: mg<br>Wenn bekannt, Medikament3: Tagesdosis: mg<br>**Wertelabels:**<br>**1 Ritalin** · **5 Concerta** · **9 Elvanse**<br>**2 Ritalin Retard** · **6 Strattera (Atomoxetin)** · **10 MPH**<br>**3 Medikinet** · **7 Equasym** · **11 MPH Retard**<br>**4 Medikinet Retard** · **8 Equasym Retard**		
VI.d	☐1 Psychoedukation & Beratung	☐0 **Nein**	☐1 **Ja**

VI.e/f	☐1 Psychotherapie ☐0 **Nein** ☐1 **Ja**
	Wenn bekannt, therapeutische Ausrichtung:<br>**<u>Wertelabels:</u>**<br>**1 Verhaltenstherapie**<br>**2 Psychoanalyse**<br>**3 Tiefenpsychologie**<br>**4 Systemische Therapie**<br>**5 Andere**<br>Wenn „Andere“, dann Angabe hier eintragen: **Freitext**
VI.g	☐1 Diagnostische Abklärung ☐0 **Nein** ☐1 **Ja**
VI.h	☐1 Sonstiges (Bitte beschreiben):**Freitext**

VERSORGUNG BEI IHNEN

1.-24. Termin am:	Dauer (in Min.)	☐ Patient	☐ Eltern	☐ Andere

VIII	Wird es weitere Termine bzgl. der ADHS- Symptomatik geben? ☐0 **Nein** (zu VIII.a-g) ☐1 **Ja** (nächste Seite)
VIII.a-g	Bitte begründen Sie die Beendigung der Behandlung (Mehrfachnennungen möglich):<br>☐a regulär beendet **(a)**<br>☐b Behandlung nicht wirksam **(b)**<br>☐c in alternative Behandlung überwiesen **(c)**<br>☐d Umzug/mangelnde Erreichbarkeit der Familie **(d)**<br>☐e Abbruch durch mich **(e)**<br>☐f Abbruch durch Eltern/Patient **(f)**<br>☐g Sonstiges (zu VIII.h)
VIII.h	**Sonstiges**: Bitte beschreiben: **Freitext**

DIAGNOSTISCHE PHASE
Angaben zu den vergangenen 3 Monaten

01	Wurden diagnostische Maßnahmen hinsichtlich der Abklärung von ADHS oder anderer psychischer Verhaltensauffälligkeiten durchgeführt?<br>☐0 **Nein (weiter zu 08)**<br>☐1 **Ja, dafür habe ich verwiesen (weiter zu 03)**<br>☐2 **Ja, von mir selbst (weiter zu 02)**<br>☐3 **Ja, von mir selbst und verwiesen (1 und 2)**
02	Wie viel Zeit haben Sie für die Diagnosestellung aufgewendet: _________ Minuten
03	Wenn Sie zu diagnostischen Zwecken verwiesen haben, erfolgte dies:<br>☐1 intern oder ☐2 extern<br>Bitte geben Sie die Berufsgruppe des Kollegen an: (drei Antwortmöglichkeiten kodierbar)<br>**<u>Wertelabels:</u>**<br>**1 Mediziner** **6 Ergotherapeut**<br>**2 Psychiater** **7 Logopäde**<br>**3 Psychologe** **8 Motopäde**<br>**4 Psychotherapeut** **9 Heilpädagoge**<br>**5 Pädagoge** **10 Andere**

04a/b	Welche Inhalte/Themen wurden im Rahmen von Exploration & Anamnese abgeklärt bzw. waren Ihnen zu diesem Zeitpunkt bekannt?

	durch Sie	durch Andere (sofern bekannt)
	mit Eltern:<br>**Wertelabels:**<br>**1 Anamnese ADHS-Symptomatik**<br>**2 Anamnese Komorbiditäten**<br>**3 Anamnese ADHS-Entwicklung**<br>**4 Anamnese Meilensteine kindlicher Entwicklung**<br>**5 Anamnese Risikofaktoren ADHS**<br>**6 Exploration/Anamnese aktueller schulischer Leistungen, Entwicklungsstand/ IQ**	mit Eltern:<br>**Wertelabels:**<br>**1 Anamnese ADHS-Symptomatik**<br>**2 Anamnese Komorbiditäten**<br>**3 Anamnese ADHS-Entwicklung**<br>**4 Anamnese Meilensteine kindlicher Entwicklung**<br>**5 Anamnese Risikofaktoren ADHS**<br>**6 Exploration/Anamnese aktueller schulischer Leistungen, Entwicklungsstand/ IQ**

Hinweise:

Zu 1a/b: **Punkt** für Formulierungen wie „Problematik", „Probleme", „Symptomatik" und „V.a. ADHS"
Kein Punkt (hier) für Formulierung **"Problemgenese"**, da dies schon wieder 3 a/b
Kein Punkt für Angaben wie „Anamnese" oder „Exploration"
Zu 2 a/b: **Punkt** für „oppositionelle Verhaltensweisen", „Ängste" und „Sprachentwicklungsstörung /-verzögerung"
Kein Punkt für Formulierungen wie „halte sich nicht an Regeln"
Zu 3a/b: **Punkt** für „Vorbehandlungen", „Behandlungs- bzw. Krankheitsgeschichte" „bisherige Versuche" und „Problemgenese" (Formulierungen „Problematik" oder Probleme hingegen bei 1a/b)
Zu 5a/b: **Punkt** für Formulierungen „Risikofaktoren ADHS", „Familie" wie auch „Eigenanamnese"
Letzteres allerdings wirklich nur, wenn es bei den Eltern benannt wurde.
Zu 6a/b: **Punkte** für Formulierungen „aktuelle Entwicklung" oder Angabe des Wortes „Schule" (reicht aus)

Allgemeine Hinweise:

1. **Kein Punkt** für die alleinige Berufsbezeichnung des Kollegen in Spalte „durch Andere"
2. Sollten hier Inhalte benannt sein, die an anderer Stelle Punkte geben, werden diese **nicht** umgebucht!!
Beispiel: „aufrechterhaltende Faktoren". Da eine reine Exploration derselben etwas anderes ist, als mittels Interventionen an deren Minimierung zu arbeiten
3. Die Benennung des Einsatzes der Kohns-Bögen gibt an dieser Stelle die volle Punktzahl

04c/d	mit Patienten:	mit Patienten:

Hinweise:

Punkt für „s.o." sofern bei Eltern sinnvolle Angaben
Kein Punkt bei Eltern gutschreiben, wenn nur an dieser Stelle die Abklärung der Kernsymptome erwähnt wird.
Kein Punkt für die alleinige Berufsbezeichnung des Kollegen in Spalte „durch Andere"

04e/f	mit Anderen	mit Anderen:

Hinweise:

Punkt für „s.o." sofern bei Eltern sinnvolle Angaben
Kein Punkt bei Eltern gutschreiben, wenn nur an dieser Stelle die Abklärung der Kernsymptome erwähnt wird.
Kein Punkt für die alleinige Berufsbezeichnung des Kollegen in Spalte „durch Andere"

05	Wurden standardisierte Fragebogenverfahren eingesetzt?

- ☐0 **Nein (weiter zu 06)**
- ☐1 **Ja, dafür habe ich verwiesen (weiter zu 05 b, d, f)**
- ☐2 **Ja, von mir selbst (weiter zu 05 a, c, e)**
- ☐3 **Ja, von mir selbst und verwiesen (1 und 2)**

	durch Sie	durch Andere (sofern bekannt)
05a/b	mit Eltern:<br>**1 FB ADHS Eltern durch Versorger**<br>**2 FB Komorbiditäten Eltern durch Versorger**	mit Eltern:<br>**1 FB ADHS Eltern durch Andere**<br>**2 FB Komorbiditäten Eltern durch Andere**

<table>
<tr><td></td><td colspan="2"><u>Hinweise:</u>
Punkt für die alleinige Angabe „DISYPS II“ bei „FBB ADHS“
Keine Übertragung von Punkten zu Diagnostik 4: „Abklärung von Komorbiditäten“, auch wenn ein entsprechender Fragebogen benannt wird
Wenn nicht klar ersichtlich ist, wer den Fragebogen tatsächlich erhalten hat (Eltern, Patient oder Lehrer) dann wird die Spalte in der es steht, streng eingehalten und entsprechend gerated. Sollte klar ersichtlich sein (z.B. durch Angaben im Vorbefund o.ä.) wer welche Fragebogen erhalten hat, wird es natürlich korrigiert.
Kein Punkt für die alleinige Berufsbezeichnung des Kollegen in Spalte „durch Andere“</td></tr>
<tr><td>05c/d</td><td>mit Patienten:
1 FB ADHS Patient durch Versorger
2 FB Komorbiditäten Patient durch Versorger</td><td>mit Patienten:
1 FB ADHS Patient durch Andere
2 FB Komorbiditäten Patient durch Anderen</td></tr>
<tr><td></td><td colspan="2"><u>Hinweise:</u>
Punkt für „s.o.“ sofern bei Eltern sinnvolle Angaben
Weitere Hinweise: siehe obige Hinweis-Box</td></tr>
<tr><td>05e/f</td><td>mit Anderen:
1 FB ADHS Andere durch Versorger
2 FB Komorbiditäten Andere durch Versorger</td><td>mit Anderen:
1 FB ADHS Andere durch Andere
2 FB Komorbiditäten Andere durch Andere</td></tr>
<tr><td></td><td colspan="2"><u>Hinweise:</u>
Punkt für „s.o.“ sofern bei Eltern/Patient sinnvolle Angaben
Weitere Hinweise: siehe obige Hinweis-Box</td></tr>
<tr><td>06</td><td colspan="2">Wurden weitere psychologische Testverfahren eingesetzt?
☐0 Nein (weiter zu 07)
☐1 Ja, dafür habe ich verwiesen (weiter zu 06 b, d, f)
☐2 Ja, von mir selbst (weiter zu 06 a, c, e)
☐3 Ja, von mir selbst und verwiesen (1 und 2)</td></tr>
<tr><td>06a/b</td><td>durch Sie
mit Eltern:</td><td>durch Andere (sofern bekannt)
mit Eltern:</td></tr>
<tr><td></td><td colspan="2"><u>Hinweis:</u>
Punkt für die Angabe „Videogestützte Verhaltensbeobachtung“</td></tr>
<tr><td>06c/d</td><td>mit Patienten:</td><td>mit Patienten:</td></tr>
<tr><td></td><td colspan="2"><u>Hinweise:</u>
Punkt bei Angaben Coloured Progressives Matrices (CPM) und Mann-Zeichen-Test (MZT)
Kein Punkt bei alleiniger Nennung projektiver o.ä. Verfahren ohne Angabe einer Intelligenzdiagnostik</td></tr>
<tr><td>06e/f</td><td>mit Anderen:</td><td>mit Anderen:</td></tr>
<tr><td></td><td colspan="2"><u>Hinweis:</u>
Punkt für die Angabe „Videogestützte Verhaltensbeobachtung“</td></tr>
<tr><td>07</td><td colspan="2">Wurden körperliche Untersuchungen am Patienten durchgeführt?
☐0 Nein (weiter zu 08)
☐1 Ja, dafür habe ich verwiesen (weiter zu 07b)
☐2 Ja, von mir selbst (weiter zu 07a)
☐3 Ja, von mir selbst und verwiesen (1 und 2)</td></tr>
<tr><td>07a/b</td><td>durch Sie
1 Körperliche Untersuchung durch Versorger</td><td>durch Andere (sofern bekannt)
1 Körperliche Untersuchung durch Andere</td></tr>
</table>

	**Hinweise:**<br><br>**Punkt** für die Angabe „Allgemeinmediziner“<br>**Punkt** für Angabe „U10“<br>Sollten an dieser Stelle Untersuchungen wie EEG, EKG, usw. aufgeführt sein, bei den Angaben zur medikamentösen Einstellung allerdings nicht mehr, wir das korrigiert und entsprechend bewertet
08a-e	F ____________ | Schweregrad: ☐1 leicht ☐2 mittel ☐3 schwer
09	Gibt es weitere diagnostische Maßnahmen / Verfahren, die Sie bei diesem Patienten für sinnvoll erachtet hätten? ☐0 Nein (zu 10) ☐1 **Ja** (zu 09a)
09a	Bitte Maßnahmen beschreiben: **Freitext - wenn folgende Antworten genannt, dann kodieren**<br><br>**Wertelabels:**<br><br>**1 Finanzmangel**<br>**2 Zeitmangel**<br>**3 Kooperationsmangel**<br>**4 Kenntnismangel**<br>**5 Patientengründe**<br>**6 Organisatorische Gründe**<br><br>**Weitere Barrieren Diagnostik: Freitext**
	**Hinweise:** **SIEHE GRUNDREGELN FÜR DIE EINGABE (1.3)**

INDIKATIONSSTELLUNG
Angaben zu den vergangenen 3 Monaten

Bei dem Patienten bestand nach Abschluss der Diagnostik eine Indikation für:

10a	Pharmakotherapie	☐0 Nein	☐1 primär	☐2 ggf. später
10b	Psychoedukation & Beratung	☐0 Nein	☐1 primär	☐2 ggf. später
10c	Psychotherapie	☐0 Nein	☐1 primär	☐2 ggf. später
10d	Sonstiges	☐0 Nein	☐1 primär	☐2 ggf. später

Bitte beschreiben: **Freitext**

	In den vergangenen 3 Monaten wurde bereits eine Behandlung durch Sie begonnen/eingeleitet	bei Ihnen	bei Anderen (sofern bekannt)	
			intern (eigene Praxis/Einrichtung)	extern (andere Praxis/Einrichtung)
11a	Pharmakotherapie	☐1 Ja	☐2 Ja	☐3 Ja
11b	Psychoedukation & Beratung	☐1 Ja	☐2 Ja	☐3 Ja
11c	Psychotherapie	☐1 Ja	☐2 Ja	☐3 Ja
11d	Sonstiges	☐1 Ja	☐2 Ja	☐3 Ja

Bitte beschreiben: **Freitext**

Hinweise:

Zu 10 & 11: Sollten die hier getätigten Kreuzchen in **eindeutigem** Widerspruch zu den weiteren Angaben stehen (z.B. in Freitexten), sollen Korrekturen vorgenommen werden. Beispiele: Indikation/Einleitung einer Pharmakotherapie mit „0“ kodiert, aber im Pharmako-Kapitel Angaben zu begonnener Medikation gemacht oder Angaben zu Bei Ihnen/Intern/Extern widersprüchlich zur übrigen Dokumentation.

Zu 11: Bei Mehrfachangaben zu bei Ihnen/intern/extern muss in den entsprechenden Kapiteln geprüft werden, welche Angaben der Versorger bei „durch Sie“ bzw. „durch Andere“ gemacht hat. **Keine Doppelkodierung!**

<table>
<tr><td>12

12a</td><td>Wurde zunächst ein anderer Behandlungsschritt eingeleitet, als nach Ihrer diagnostischen Entscheidung primär indiziert war? ☐0 Nein (zu 13) ☐1 Ja (zu 12a)

Bitte Maßnahmen beschreiben: Freitext - wenn folgende Antworten genannt, dann kodieren

<u>Wertelabels:</u>

1 Finanzmangel
2 Zeitmangel
3 Kooperationsmangel
4 Kenntnismangel
5 Patientengründe
6 Organisatorische Gründe

Weitere Barrieren Indikation: Freitext</td></tr>
<tr><td></td><td><u>Hinweise</u>: SIEHE GRUNDREGELN FÜR DIE EINGABE (1.3)</td></tr>
<tr><td colspan="2">PSYCHOEDUKATION UND BERATUNG</td></tr>
<tr><td>13</td><td>Wurden psychoedukative oder beratende Inhalte/Themen bereits erörtert?

☐0 Nein (weiter zu 14)
☐1 Ja, dafür habe ich verwiesen (weiter zu 13 b, d, f)
☐2 Ja, von mir selbst (weiter zu 13 a, c, e)
☐3 Ja, selbst und verwiesen (1 und 2)</td></tr>
<tr><td>13a/b</td><td>
<table>
<tr><td>durch Sie
mit Eltern:

<u>Wertelabels:</u>

1 Erfassung subjektiver Störungskonzepte
2 Infovermittlung zu ADHS
3 Erarbeitung gemeinsames Störungskonzept
4 Erarbeitung gemeinsames Behandlungskonzept
5 Erkennen/Bearbeiten von Sorgen bzgl. ADHS
6 Aufklärung über allgemeine Erziehungsstrategien
7 Analyse/Korrektur problemaufrechterhaltender Bedingungen
8 Aufbau bzw. Stärkung von Änderungsmotivation
9 Elterntraining durchgeführt</td>
<td>durch Andere (sofern bekannt)
mit Eltern:

<u>Wertelabels:</u>

1 Erfassung subjektiver Störungskonzepte
2 Infovermittlung zu ADHS
3 Erarbeitung gemeinsames Störungskonzept
4 Erarbeitung gemeinsames Behandlungskonzept
5 Erkennen/Bearbeiten von Sorgen bzgl. ADHS
6 Aufklärung über allgemeine Erziehungsstrategien
7 Analyse/Korrektur problemaufrechterhaltender Bedingungen
8 Aufbau bzw. Stärkung von Änderungsmotivation
9 Elterntraining durchgeführt</td></tr>
</table>
<u>Hinweise:</u>

Zu 1a/b: Kein Punkt an dieser Stelle für Bezeichnung „Ursachen“, sondern Punkt bei 3a/b!
Zu 2a/b: Punkt für „Ursachen“/„Ätiologie“ (auch wenn das bedeuten könnte, Versorger hat einseitig vermittelt
Zu 4a/b: Punkt nur für Formulierungen wie „Therapiemöglichkeiten“ , „Option der Medikation“, o.ä.
Kein Punkt für „Umgang mit ADHS“ oder „Therapie“, da zu unspezifisch
Zu 5a/b: Punkt für Formulierung „Folgen“ (oder sobald erkennbar, dass emotional oder kognitiv spezifische Befürchtungen zu Folgen der Symptomatik Thema waren
Kein Punkt für „Unterstützung bei ADHS“, da zu unspezifisch
Punkt für Angabe „Nachteilsausgleich“ oder „Bescheinigung für Schule“
Zu 6a/b: Punkt pauschal geben, sobald Versorger konkrete Interventionen in den Bereichen Psychoedukation oder Psychotherapie benennt
Punkt wenn im Themenbereich Psychotherapie „Regeln“ oder „Konsequenzen“ benannt
Zu 7a/b: Punkt für „Teufelskreis“, „Token-System“, „Verstärker“ o. ä. strukturierungsspezifische Interventionen
Kein Punkt bei allgemeinen Formulierungen wie „Strukturierung des Alltags“
Kein Punkt für Formulierung „mehr Konsequenz und Anleitung zu mehr Selbstständigkeit
Zu 8a/b: Punkt für „Stärken“ bzw. „Ressourcen“ (auch, wenn in Bereich Psychotherapie erwähnt)
Kein Punkt wenn klar ersichtlich, dass hiermit gemeint ist, dass die Ressourcen des Patienten mit den Eltern besprochen wurden. Dann aber Punkt bei Patient (5) vergeben
Zu 9a/b: Punkt nur vergeben, wenn außer dem Wort „Elterntraining“ bzw. der Benennung konkreter Elterntraining-Programme keine detailreicheren Angaben gemacht werden. Sobald die Angaben spezifischer sind, werden diese unter 1-8 verschlüsselt</td></tr>
</table>

Allgemeine Hinweise:

1. **Kein Punkt** für die alleinige Berufsbezeichnung des Kollegen in Spalte „durch Andere"
2. Formulierungen wie „Achtsamkeitsübungen" und „Progressive Muskelentspannung" müssen leider unbepunktet bleiben, da dies in den Leitlinien nicht formuliert ist.

13c/d	mit Patient:<br>**Wertelabels:**<br>**1 Erfassung subjektiver Störungskonzepte**<br>**2 Infovermittlung zu ADHS**<br>**3 Erarbeitung gemeinsames Störungskonzept**<br>**4 Erarbeitung gemeinsames Behandlungskonzept**<br>**5 Aufbau und Stärkung von Änderungsmotivation**	mit Patient:<br>**Wertelabels:**<br>**1 Erfassung subjektiver Störungskonzepte**<br>**2 Infovermittlung zu ADHS**<br>**3 Erarbeitung gemeinsames Störungskonzept**<br>**4 Erarbeitung gemeinsames Behandlungskonzept**<br>**5 Aufbau und Stärkung von Änderungsmotivation**

Hinweise:

Zu 1a/b: **Kein Punkt** an *dieser* Stelle für Bezeichnung „Ursachen", sondern **Punkt bei 3a/b**
Zu 2a/b: **Punkt** für „ADS-Schulung"
Zu 3a/b: **Punkt** für „Ursachen" (**Punkt** sobald annähernd genannt, selbst wenn Formulierung implizieren könnte, der Versorger habe einseitig vermittelt
Zu 5a/b: **Punkt** für „Stärken" bzw. „Ressourcen" (auch, wenn in Bereich Psychotherapie erwähnt)
Punkt für Betonung der Entwicklung *eigener* Lösungsstrategien
Punkt für Formulierung „Autonomieentwicklung"
Punkt für Formulierung „SORC"

Allgemeine Hinweise:

1. **Punkt** für „s.o." sofern bei Eltern sinnvolle Angaben
2. **Kein Punkt** für die alleinige Berufsbezeichnung des Kollegen in Spalte „durch Andere"
3. Formulierungen wie „Achtsamkeitsübungen" und „Progressive Muskelentspannung" müssen leider unbepunktet bleiben, da dies in den Leitlinien nicht formuliert ist.
4. Dass es zudem hier nicht möglich ist, konkrete Interventionen zu bepunkten, muss leider hingenommen werden!

13e/f	mit Anderen:<br>**Wertelabels:**<br>**1 Erfassung subjektiver Störungskonzepte**<br>**2 Infovermittlung zu ADHS**<br>**3 Aufklärung über allgemeine Erziehungsstrategien**<br>**4 Analyse/Korrektur problemaufrechterhaltender Bedingungen**<br>**5 Aufbau bzw. Stärkung von Änderungsmotivation**	mit Anderen:<br>**Wertelabels:**<br>**1 Erfassung subjektiver Störungskonzepte**<br>**2 Infovermittlung zu ADHS**<br>**3 Aufklärung über allgemeine Erziehungsstrategien**<br>**4 Analyse/Korrektur problemaufrechterhaltender Bedingungen**<br>**5 Aufbau bzw. Stärkung von Änderungsmotivation**

Hinweise:

Zu 1a/b: **Kein Punkt** an *dieser* Stelle für Bezeichnung „Ursachen", sondern **Punkt bei 13a/b (3)**
Zu 3a/b: **Punkt** für „Ursachen" (**Punkt** sobald annähernd genannt, selbst wenn Formulierung implizieren könnte, der Versor ger habe einseitig vermittelt
Punkt für „unkontrollierte Impulsreaktionen mit Schule besprochen"
Zu 5a/b: **Punkt** für „Stärken" bzw. „Ressourcen" (auch, wenn in Bereich Psychotherapie erwähnt).
Punkt für „s.o." sofern bei Eltern/Patient sinnvolle Angaben
Kein Punkt für die alleinige Berufsbezeichnung des Kollegen in Spalte „durch Andere"

14	Gibt es (weitere) psychoedukative/ beratende Inhalte/Themen, die Sie für sinnvoll erachtet hätten?	☐$_0$ Nein (zu 15) ☐$_1$ Ja (zu 14a)
14a	Bitte Maßnahmen beschreiben: **Freitext - wenn folgende Antworten genannt, dann kodieren**<br>**Wertelabels:**<br>**1 Finanzmangel**<br>**2 Zeitmangel**<br>**3 Kooperationsmangel**<br>**4 Kenntnismangel**<br>**5 Patientengründe**<br>**6 Organisatorische Gründe**	
	**Weitere Barrieren Psychoedukation: Freitext**	

Hinweise: **SIEHE GRUNDREGELN FÜR DIE EINGABE (1.3)**

PHARMAKOTHERAPIE	
15	Wird der Patient zurzeit medikamentös wegen ADHS behandelt?<br>☐0 **Nein (weiter zu 15a)**<br>☐1 **Ja, dafür habe ich verwiesen (weiter zu 15b)**<br>☐2 **Ja, von mir selbst (weiter zu 15c)**<br>☐3 **Ja, selbst und verwiesen (1 und 2)**
15a	Wenn zurzeit keine medikamentöse Behandlung erfolgt, machen Sie hierzu bitte genauere Angaben:
	☐0 **Nein, noch nie – soweit bekannt – (weiter zu 23)**<br>☐1 **Behandlungsversuch innerhalb der vergangenen 3 Monate beendet (weiter zu 15c)**<br>☐2 **Aktueller Auslassversuch (weiter zu 15c)**
15b	Wenn Sie verwiesen haben, geben Sie bitte an, ob Ihnen von anderer Stelle Informationen übermittelt wurden: ☐0 Nein (zu 23) ☐1 Ja (zu 15c)
15c	Geben Sie die aktuelle oder letzte Medikation und Dosierung an (Angabe für bis zu 3 Medikamente)<br>Medikament (1-3): Tagesdosis: mg<br><br>**Wertelabels:**<br>**1 Ritalin** · **5 Concerta** · **9 Elvanse**<br>**2 Ritalin Retard** · **6 Strattera (Atomoxetin)** · **10 MPH**<br>**3 Medikinet** · **7 Equasym** · **11 MPH Retard**<br>**4 Medikinet Retard** · **8 Equasym Retard**<br><br>Weitere Informationen: **Freitext**
	**Hinweis:**<br>Sofern sich hier eine Angabe dahingehend finden sollte, dass der Versorger die Eindosierung anhand des Körpergewichts des Patienten vorgenommen hat, erhält er dafür einen Punkt bei 17a_2, sofern er diesen nicht schon aufgrund der dortigen Angaben erhalten hat.

Die folgenden Fragen müssen Sie nur beantworten, wenn Sie selbst eine Pharmakotherapie beim Patienten

- **bereits begonnen haben,**
- **oder einen Behandlungsversuch beendet haben**
- **oder derzeit ein Auslassversuch durchgeführt wird.**

16	Bitte geben Sie die Anzahl der Kontakte an (z.B. direkt, telefonisch), in denen die Ermittlung der optimalen Dosierung für den Patienten Thema war: ________ Kontakte
17	Wurden spezifische körperliche Parameter überprüft? ☐0 Nein ☐1 Ja (zu 17a)

17a	Welche körperlichen Parameter und zu welchem(n) Zeitpunkt(en):<br>**Sofern Nennung mehrerer Termine „Kontrolluntersuchungen erfolgt" kodieren**<br>**<u>Wertelabels:</u>**<br>**1 Körpergröße**<br>**2 Gewicht**<br>**3 Blutdruck/Pulsfrequenz**<br>**4 And. spezifische körperl. Untersuchungen (z.B. Blutbild, EEG, EKG)**<br>**5 Welche anderen körperlichen Untersuchungen: Freitext**
	**<u>Hinweise:</u>**<br>Zu 1: **Punkt** für Formulierung „Körperstatus" (s. auch „zu 2")<br>Zu 2: **Punkt** für Formulierung „Körperstatus" (s. auch „zu 1")<br>Zu 4: An dieser Stelle werden ausschließlich „spezifische" Untersuchungen kodiert! Tauchen hier allgemeinere Bezeichnungen wie „körperliche Untersuchungen", Hör- oder Sehtests auf, wird die Angabe verschoben und gibt dann im Abschnitt Diagnostik unter bei **7a/b** einen Punkt.<br>Zu 5: Sollte gemäß Angaben keine Pharmakotherapie durchgeführt werden, es wurden aber EEG, BB oder EKG erstellt, dann wird der Freitext eingetragen, die Messung aber nicht unter 4 gerated.
18	Wurden im Rahmen der medikamentösen Behandlung spezifische Inhalte/Themen abgeklärt? ☐$_0$ Nein ☐$_1$ Ja (zu 18 a-c)
18a	mit den Eltern:**Spezifische Themen/ Inhalte bez. Medikation mit Eltern besprochen (Ja/Nein)**
18b	mit den Patienten: **Spezifische Themen/ Inhalte bez. Medikation mit Patient besprochen (Ja/Nein)**
18c	mit Anderen: **Spezifische Themen/ Inhalte bez. Medikation mit Anderen besprochen (Ja/Nein)**
19	Wurden hierbei standardisierte Fragebögen eingesetzt? ☐$_0$ Nein ☐$_1$ Ja (zu 19a)
19a	Bitte beschreiben:<br>**<u>Wertelabels:</u>**<br>**1 Wurde CM_ADHS eingesetzt**<br>**2 Wurde ADHS-TAP eingesetzt**<br>**3 Wurde NW-ADHS eingesetzt**<br>**4 Wurde FBB-ADHS eingesetzt**<br>**5 Wurden andere Fragebögen eingesetzt**<br>**Sonstige Fragebögen: Freitext**
20a	Welche Informationen haben Sie im Rahmen der medikamentösen Behandlung vermittelt?<br>an die Eltern:<br>**<u>Wertelabels:</u>**<br>**1 Aufklärung der Eltern über Medikation/Wirkweise**<br>**2 Exploration der Wünsche/ Ängste der Eltern bez. Medikation**<br>**3 Besprechung der Medikamenteneinnahme mit Eltern**<br>**4 Zielsymptome mit Eltern besprochen**<br>**5 Eventuelle Nebenwirkungen mit Eltern besprochen**<br>**6 Besprechen weiterer Themen bez. Medikation mit Eltern**
	**<u>Hinweise:</u>**<br>Zu 2: **Punkt** sobald annähernd genannt, auch wenn Formulierung implizieren könnte, Versorger habe einseitig vermittelt<br>Zu 4: **Punkt** ausschließlich für die konkrete Bezeichnung „Zielsymptome" oder Beschreibung differenzierter Verhaltensweisen, die durch Medis beeinflusst werden sollen<br>**Kein Punkt** für Formulierung „Wirkung", da zu allgemein
20b	an Patienten:<br>**<u>Wertelabels:</u>**<br>**1 Aufklärung des Patienten über Medikamentenwirkung**<br>**2 Aufklärung des Patienten über mögliche Nebenwirkungen**<br>**3 Besprechen weiterer Themen bez. Medikation mit Patienten**
20c	an Andere: **Information an Andere über Medikation (Ja/Nein)**

21	Bitte beschreiben Sie, wie die derzeitige Tagesdosis ermittelt wurde: **Freitext**
22	Wurde innerhalb der vergangenen 3 Monate ein Auslassversuch durchgeführt? ☐$_0$ Nein (zu 23) ☐$_1$ Ja (zu 22a)
22a	Bitte beschreiben Sie den Ablauf: **Freitext**
23	Gibt es (weitere) Maßnahmen/Verfahren, die Sie im Zusammenhang mit der medikamentösen Behandlung für sinnvoll erachtet hätten? ☐$_0$ Nein ☐$_1$ Ja (zu 23a)
23a	Nennen Sie uns bitte kurz die betreffenden Inhalte/Themen und die Gründe warum diese nicht besprochen wurden/ werden konnten.<br>Bitte Maßnahmen beschreiben: **Freitext - wenn folgende Antworten genannt, dann kodieren**<br>**<u>Wertelabels:</u>**<br>**1 Finanzmangel**<br>**2 Zeitmangel**<br>**3 Kooperationsmangel**<br>**4 Kenntnismangel**<br>**5 Patientengründe**<br>**6 Organisatorische Gründe**<br>**Weitere Barrieren Pharmakotherapie: Freitext**
	**<u>Hinweise</u>: SIEHE GRUNDREGELN FÜR DIE EINGABE (1.3)**

PSYCHOTHERAPIE

24	Wird der Patient zurzeit bzgl. der ADHS-Symptomatik psychotherapeutisch behandelt?<br>☐$_0$ **Nein**<br>☐$_1$ **Ja, dafür habe ich verwiesen**<br>☐$_2$ **Ja, von mir selbst**<br>☐$_3$ **Ja, selbst und verwiesen (1 und 2)**
	**<u>Hinweis:</u>**<br>Sofern ersichtlich wird, dass Versorger keine richtliniengetreue Psychotherapie durchgeführt hat (bspw. hier ergotherapeutische Interventionen genannt oder ausschließlich Inhalte, die unter Psychoedukation kodiert werden, dann Korrektur der Kreuzchen zu „0“ = Nein und ggf. Anpassung der Angaben bei Indikationsstellung „selbst durchgeführt“!
25	Wird der Patient wegen komorbider Symptomatiken psychotherapeutisch behandelt?<br>☐$_0$ **Nein**<br>☐$_1$ **Ja, dafür habe ich verwiesen. (weiter zu 26)**<br>☐$_2$ **Ja, von mir selbst. (weiter zu 27)**<br>☐$_3$ **Ja, selbst und verwiesen (1 und 2).**
	**<u>Hinweis:</u>**<br>Sofern ersichtlich wird, dass Versorger keine richtliniengetreue Psychotherapie durchgeführt hat (bspw. hier ergotherapeutische Interventionen genannt oder ausschließlich Inhalte, die unter Psychoedukation kodiert werden, dann Korrektur der Kreuzchen zu „0“ = Nein und ggf. Anpassung der Angaben bei Indikationsstellung „selbst durchgeführt“!

Haben Sie die beiden letzten Fragen mit „Nein“ beantwortet?
Dann sind Sie fast fertig (weiter zu 30)!

Aber jetzt ist es auch für alle anderen gleich geschafft ☺

26<br><br>26a	Wenn Sie verwiesen haben, geben Sie bitte an, ob Ihnen Informationen von anderer Stelle übermittelt wurden: ☐$_0$ Nein ☐$_1$ Ja (zu 26a)<br><br>Welche Informationen zur Therapie: **Freitext - wenn folgende Antworten genannt, dann kodieren**<br><br>**<u>Wertelabels:</u>**<br><br>**1 Information über Störungsmodell durch Andere**<br>**2 Information über gemeinsamen Behandlungsplan durch Andere**<br>**3 Psychoedukation und Beratung durch Andere**<br>**4 Information über bestimmte Therapiemodule durch Andere**<br>**5 Bedeutung positiver Eltern-Kind-Beziehung durch Andere**<br>**6 Sonstige Informationen zur Therapie durch Andere**
	**<u>Hinweis:</u>**<br><br>Sofern ersichtlich, dass für die Psychotherapie **intern** verwiesen wurde, Angaben korrigieren und raten als hätte Versorger Psychotherapie selbst durchgeführt!
**Die folgenden Fragen müssen Sie nur beantworten, wenn <u>Sie selbst</u> eine entsprechende Behandlung bei diesem Patienten <u>bereits begonnen</u> haben.**	
27	Wurden im Rahmen der Psychotherapie spezifische Inhalte/Themen oder Ziele besprochen? ☐$_0$ Nein ☐$_1$ Ja (zu 27a-c)
27a	mit Eltern: **ADHS-spezifische Themen/Inhalte wurden mit Eltern besprochen (Ja/Nein)**
27b	mit Patienten: **ADHS-spezifische Themen/Inhalte wurden mit Patienten besprochen (Ja/Nein)**
27c	mit Anderen: **ADHS-spezifische Themen/Inhalte wurden mit Anderen besprochen (Ja/Nein)**
	**<u>Hinweis:</u>**<br><br>Werden hier bereits Interventionen, wie z.B. „Einsatz von Verstärkern" genannt, dann wird die Angabe korrigiert und entsprechend bei 28a-c gewertet
28	Wurden im Rahmen der Psychotherapie der ADHS-Symptome spezifische Interventionen durchgeführt? ☐$_0$ Nein ☐$_1$ Ja (zu 28a-c)
28a	mit Eltern:<br><br>**<u>Wertelabels:</u>**<br><br>**1 Verhaltensanalyse mit Eltern durchgeführt**<br>**2 Intensivierung Eltern-Kind-Beziehung besprochen**<br>**3 Wirkungsvolle Aufforderungen mit Eltern besprochen**<br>**4 Positive Konsequenzen mit Eltern besprochen**<br>**5 Angemessene negative Konsequenzen mit Eltern besprochen**<br>**6 Token-Modelle mit Eltern besprochen**<br>**7 Auszeiten mit Eltern besprochen**<br>**8 Elterntraining durchgeführt**
	**<u>Hinweise:</u>**<br><br>Zu 1: **Kein Punkt** für „Umgang mit Lautstärke"<br>Zu 3: **Punkt** für die Formulierung „Regeln"<br>**Kein Punkt** für Formulierung „Strukturgebung"<br>Zu 4: **Punkt** für Formulierung „Konsequenzen" & „konsequenter Erziehungsstil" (s. auch „zu 5")<br>Zu 5: **Punkt** für Formulierung „Konsequenzen" & „konsequenter Erziehungsstil" (s. auch „zu 4")<br>Zu 7: Gemeint sind hier Interventionen wie „stille Treppe" usw. und nicht die elterliche Psychohygiene<br>Zu 8: Punkt nur dann geben, wenn außer dem Wort „Elterntraining" keine detailreicheren en Angaben. Sobald die Angaben spezifischer sind, werden diese unter 1-7 verschlüsselt. Dieser Punkt wird ausschließlich **einmal** vergeben. Sofern also schon bei Psychoedukation verbucht, gibt es hier keinen weiteren Punkt.
	**<u>Allgemeine Hinweise:</u>**<br><br>**Punkt** (pauschal) nach **13a/b (7)**, wenn „Teufelskreis" genannt)<br>**Punkt** nach **13a/b (8)**, wenn hier von „Stärken bzw. Ressourcen" die Rede ist. Sollte jedoch klar ersichtlich sein, dass hiermit gemeint ist, dass die Ressourcen des Patienten mit den Eltern besprochen wurden Punkt unter **13a/b (5)**

28b	mit Patienten:<br>**<u>Wertelabels:</u>**<br>**1 Spieltraining mit Patienten durchgeführt**<br>**2 Aufmerksamkeitstraining mit Patienten durchgeführt**<br>**3 Selbstinstruktionstraining mit Patienten durchgeführt**<br>**4 Neuropsychologisches Training mit Patienten durchgeführt**<br>**5 Selbstmanagement mit Patienten durchgeführt**<br>**6 Problemlösetraining mit Patienten durchgeführt**<br>**7 Kommunikationstraining mit Patienten durchgeführt**<br>**8 Kognitive Umstrukturierung mit Patienten**<br>**9 Therapieprogramm oder Gruppentherapie durchgeführt**
	**<u>Hinweise:</u>**<br>Zu 5 & 6: Abgrenzung der beiden ist etwas schwammig, daher Einigung: Sofern die Vergabe beider Punkte nicht eindeutig vertretbar sein sollte, kein Doppelrating vornehmen, sondern sich für einen der beiden Punkte entscheiden.<br>Zu 6: **Punkt** für Angabe „soziales Kompetenztraining“ (s. auch „zu 7“)<br>Zu 7: **Punkt** für Angabe „soziales Kompetenztraining“ (s. auch „zu 6“)<br>**Punkt nach 13c/d (5)** wenn hier erwähnt, dass mit dem Patienten „Stärken bzw. Ressourcen“ besprochen
28c	mit Anderen:<br>**<u>Wertelabels:</u>**<br>**1 Verhaltensanalyse in der Schule/ im Kindergarten mit Anderen durchgeführt**<br>**2 Positive Lehrer/Erzieher/Kind-Interaktionen durchgeführt**<br>**3 Wirkungsvolle Aufforderung mit Anderen besprochen**<br>**4 Positive Konsequenzen mit Anderen besprochen**<br>**5 Angemessene neg. Konsequenzen mit Anderen besprochen**<br>**6 Token-Modelle mit Anderen besprochen**<br>**7 Auszeit durch Andere besprochen**
29	Haben Sie dabei therapeutische Manuale eingesetzt? ☐$_0$ Nein ☐$_1$ Ja (zu 29a)
29a	Bitte beschreiben: **Freitext**
30	Gibt es (weitere) Maßnahmen/Verfahren, die Sie im Zusammenhang mit der psychotherapeutischen Behandlung für sinnvoll erachtet hätten? ☐$_0$ Nein ☐$_1$ Ja (zu 30a)
30a	Bitte Maßnahmen beschreiben: **Freitext - wenn folgende Antworten genannt, dann kodieren**<br>**<u>Wertelabels:</u>**<br>**1 Finanzmangel**<br>**2 Zeitmangel**<br>**3 Kooperationsmangel**<br>**4 Kenntnismangel**<br>**5 Patientengründe**<br>**6 Organisatorische Gründe**<br>**Weitere Barrieren Psychotherapie: Freitext**
	**<u>Hinweise</u>: SIEHE GRUNDREGELN FÜR DIE EINGABE (1.3)**
