# Supplementary material for: Guideline adherence in German routine care of children and adolescents with ADHD: an observational study
Source: Eur Child Adolesc Psychiatry. 2020 May 28;30(5):757–68. doi: 10.1007/s00787-020-01559-8 (PMC8060198; doi:10.1007/s00787-020-01559-8)
Supplement: Supplementary file 3 — Supplementary material 3 (PDF 284 kb) [file 787_2020_1559_MOESM3_ESM.pdf]

Supplementary table S1

Assessment (AS): guideline adherence rates per item[a] (patient level)

	Overall (*n*=167 patients)	Paediatricians (*n*=50 patients)	Psychiatrists (*n*=81 patients)	Psychotherapists (*n*=36 patients)
**Guideline Component**	*%*	*%*	*%*	*%*
Clinical interview with parents regarding…				
1. # ADHD symptoms	64.67	62.00	74.07	47.22
2. # Coexisting conditions	32.93	36.00	29.63	36.11
3. # ADHD course	29.34	34.00	32.10	16.67
4. # Milestones of early development	32.34	32.00	35.80	25.00
5. # ADHD risk factors	50.30	56.00	53.09	36.11
6. # Current developmental level	52.69	70.00	49.38	36.11
Any of components 1. – 6.	*88.62*	*90.00*	*93.83*	*75.00*
7. # Clinical interview with patient	83.23	80.00	91.36	69.44
8. Clinical interview with others (e.g. teachers)	34.73	34.00	34.57	36.11
Use of ADHD rating scales…				
9. for parents	80.84	78.00	83.95	77.78
10. for patient [b]	77.84	86.00	71.60	80.56
11. for teachers	60.48	54.00	67.90	52.78
Use of rating scales for coexisting conditions…				
12. for parents	60.48	54.00	61.73	66.67
13. for patient [b]	79.64	86.00	75.31	80.56
14. for teachers	43.71	36.00	44.44	52.78
15. Use of psychological tests for patient	90.42	88.00	92.59	88.89
16. # Physical examination of patient	66.47	90.00	60.49	47.22
At least one of mandatory components	*96.41*	*100.00*	*98.77*	*86.11*

[a]Component variables give dichotomous information about fulfilment (coded as 0=no, 1=yes)
[b]Initially these components were rated=1 for all cases. As juvenile self-reports can only be validly assessed by adolescents and not children, the rating was changed to 0 only, if self-reports had not been used with adolescent patients
[#]Components defined as mandatory standard

Supplementary table S2

Treatment Indication (TI)[a]: guideline adherence rates per item[b] (patient level)

		Overall (*n*=167 patients)	Paediatricians (*n*=50 patients)	Psychiatrists (*n*=81 patients)	Psychotherapists (*n*=36 patients)
**Psychoeducation**		%	%	%	%
	Primary	95.81	96.00	95.06	97.22
	Later if necessary	1.80	0.00	3.70	0.00
	None	2.40	4.00	1.23	2.78
1.	Indication correct (component fulfilled)	97.01	96.00	96.30	100.00
**Pharmacotherapy**					
	Primary	47.31	50.00	40.74	58.33
	Later if necessary	29.34	16.00	41.98	19.44
	None	23.35	34.00	17.28	22.22
2a.	Indication correct (component fulfilled for TI_{OLD})	65.27	62.00	70.37	58.33
2b.	Indication correct (component fulfilled for TI_{NEW})	97.60	92.00	100.00	100.00
**Psychotherapy**					
	Primary	40.12	30.00	20.99	97.22
	Later if necessary	28.74	36.00	35.80	2.78
	None	31.14	34.00	43.21	0.00
3.	Indication correct (component fulfilled)	97.01	96.00	96.30	100.00

[a]As there is only one component for each of the three treatment options, no mandatory standard for TI was defined
[b]Component variables give dichotomous information about fulfilment (coded as 0=no, 1=yes)
[c]Component variables (1.-3.) do not represent the simple sum of all possible options listed above but result from complex combinations partially even across sections (e.g. PE and PT). Only components 1. - 3. were used in TI Index with introduction of either 2a. or 2b. for TI_{OLD} and TI_{NEW}

Supplementary table S3

Psychoeducation (PE): guideline adherence rates per item[a] (patient level)

		Overall (*n*=167 patients)	Paediatricians (*n*=50 patients)	Psychiatrists (*n*=81 patients)	Psychotherapists (*n*=36 patients)
**Guideline Component**		%	%	%	%
	Psychoeducation with parents for…				
1.	Exploring subjective health beliefs	2.99	2.00	2.47	5.56
2. #	Information about ADHD	42.51	30.00	53.09	36.11
3.	Common model of the disorder	17.37	12.00	23.46	11.11
4.	Common treatment concept	38.92	42.00	38.27	36.11
5.	Resolving worries concerning ADHD	15.57	16.00	18.52	8.33
6.	Imparting general education strategies	64.67	48.00	65.43	86.11
7.	Modifying problem-maintaining conditions	52.69	30.00	51.85	86.11
8.	Strengthening therapy motivation	10.78	18.00	7.41	8.33
Any of components 1. – 8. plus "parent training" [b]		*81.44*	*76.00*	*80.25*	*91.67*
	Psychoeducation with patient for…				
9.	Exploring subjective health beliefs	0.60	0.00	0.00	2.78
10. #	Information about ADHD	31.14	20.00	37.04	33.33
11.	Common model of the disorder	8.38	4.00	11.11	8.33
12.	Common treatment concept	22.16	14.00	28.40	19.44
13.	Strengthening therapy motivation	26.95	20.00	24.69	41.67
Any of components 9. – 13.		*52.10*	*32.00*	*59.26*	*63.89*
	Psychoeducation with others for…				
14.	Exploring subjective health beliefs	0.00	0.00	0.00	0.00
15.	Information about ADHD	7.19	4.00	4.94	16.67
16.	Imparting general education strategies	13.77	8.00	12.35	25.00
17.	Modifying problem-maintaining conditions	11.98	8.00	11.11	19.44
18.	Strengthening therapy motivation	1.20	0.00	0.00	5.56
Any of components 14. – 18.		*16.77*	*10.00*	*12.35*	*36.11*
At least one of mandatory components plus "parent training" [2]		*52.10*	*44.00*	*58.02*	*50.00*

[a]Component variables give dichotomous information about fulfilment (coded as 0=no, 1=yes)
[b]Unspecific verbalisation of "parent training" could be considered for this calculation
[#]Components defined as mandatory standard

Supplementary table S4

Pharmacotherapy (PH): guideline adherence rates per item[a] (patient level)

		Overall (*n*=45 patients)[b]	Paediatricians (*n*=20 patients)	Psychiatrists (*n*=25 patients)
**Guideline Component**		*%*	*%*	*%*
1. #	Selection of drug	97.78	95.00	100.00
2. #	Dosage	95.56	95.00	96.00
3.	At least 2 visits per drug during titration	77.78	70.00	84.00
	Specific somatic parameters of patient measured			
4. #	Height	62.22	70.00	56.00
5. #	Weight	68.89	70.00	68.00
6. #	Blood pressure/pulse	57.78	60.00	56.00
7.	Follow-up examination	40.00	45.00	36.00
	Use of rating scales during titration concerning			
8.	ADHD symptoms	17.78	35.00	4.00
9.	Side effects	11.11	20.00	4.00
10.	Documentation of titration process	8.89	15.00	4.00
	Clarified with parents			
11. #	Information about drug/effects	93.33	95.00	92.00
12.	Discussion of hopes/worries	33.33	20.00	44.00
13. #	Mode of drug application	22.22	15.00	28.00
14.	Definition of target symptoms for medication	20.00	20.00	20.00
15. #	Information about possible side effects	91.11	95.00	88.00
Any of components 11. – 15.		*93.33*	*95.00*	*92.00*
	Clarified with patient			
16. #	Information about drug/effects	66.67	60.00	72.00
17. #	Information about possible side effects	55.56	45.00	64.00
18.	Information about medication provided to others	15.56	25.00	8.00
	Determination of daily dosage			
19.	up-/down-titration/change of drug	75.56	80.00	72.00
20.	based on weight	15.56	5.00	24.00
21.	involving school in titration process	13.33	15.00	12.00
At least one of mandatory components		*100.00*	*100.00*	*100.00*

[a]Component variables give dichotomous information about fulfilment (coded as 0=no, 1=yes)
[b]Rates calculated based solely on documentations of patients treated with pharmacotherapy by the reporting Health Care Providers themselves

Supplementary table S5

Psychotherapy (PT): guideline adherence rates per item[a] (patient level)

		Overall (*n*=39 patients)[b]	Psychiatrists (*n*=6 patients)	Psychotherapists (*n*=33 patients)
**Guideline Component**		*%*	*%*	*%*
	Interventions with parents			
1. #	Behaviour analysis	15.38	16.67	15.15
2. #	Facilitating positive child-parent relationship	23.08	33.33	21.21
3. #	Communicating effective requests	58.97	33.33	63.64
4.(#)	Applying natural consequences	48.72	50.00	48.48
5.(#)	Token economy	43.59	0.00	51.52
6.(#)	Timeout	5.13	0.00	6.06
Any of components 1. – 6. plus "parent training" [c]		*87.18*	*66.67*	*90.91*
	Interventions with patient			
7.	Play/communication Training/Cognitive Restructuring	28.21	0.00	33.33
8.	Self-Instruction/Attention Training	71.79	33.33	78.79
9.	Neuropsychological Training	0.00	0.00	0.00
10.	Self-management	33.33	16.67	36.36
Any of components 7. – 10. plus specific programme[d]		*79.49*	*50.00*	*84.85*
11.	Interventions with others	15.38	0.00	18.18
12.	Use of specific therapeutic manuals	79.49	66.67	81.82
At least one of mandatory components plus "parent training"[3]		*97.44*	*83.33*	*100.00*

[a]Component variables give dichotomous information about fulfilment (coded as 0=no, 1=yes)
[b]Rates calculated based solely on documentations of patients treated with psychotherapy by the reporting Health Care Providers themselves
[c]Unspecific verbalisation of "parent training" could be considered for this calculation; [4] Mention of a specific treatment program (e.g. SAVE, THOP) could be considered for this calculation
[#]Components defined as mandatory standard
[(#)]Mandatory standard component: alternatively one out of components 4.-6

Supplementary table S6

Characteristics of HCPs[a] participating in first phase only (1st) and those participating in both phases (1st & 2nd)

Characteristics (interval-scaled)	Phases[b]	*n*	*M*	*SD*	*t*	*p*
Age (years)	1st<br>1st & 2nd	290<br>73	50.00<br>48.84	9.40<br>9.51	0.94	.347
Time in current position (years)	1st<br>1st & 2nd	290<br>73	9.71<br>10.30	7.33<br>7.74	-0.61	.543
ADHD expertise (years)	1st<br>1st & 2nd	290<br>73	15.40<br>13.97	8.21<br>7.72	0.41	.179
**Characteristics (dichotomous)**		*n*	*%*			*p*
Sex (male)	1st<br>1st & 2nd	137<br>30	47.24<br>41.10			.020[c]
Qualification completed[d]	1st<br>1st & 2nd	270<br>61	93.10<br>83.56			≤.001[c]
Further ADHD training	1st<br>1st & 2nd	136<br>36	46.90<br>49.32			.224[c]
Additional ADHD contract[e]	1st<br>1st & 2nd	77<br>28	26.55<br>38.36			≤.001[c]

[a]HCPs = Health Care Providers
[b]1st phase: global report about ADHD routine care via interview, 2nd phase: individual-based documentation of ADHD routine care
[c]Binomial tests were run for dichotomous variables by inserting the observed proportion of “1st & 2nd” group as test proportion for participants of “first phase only”
[d]Specialised medical certificate (paediatrician or child and adolescent psychiatrist) or licence to practise (child and adolescent psychotherapist)
[e]Participation in programmes offered by several Health Insurance Companies to support adherent care with additional financial means

Supplementary table S7

Scores of globally reported GA: Comparison of HCPs[a] participating in first phase only (1st) with those participating in both phases (1st & 2nd)

**GA Scores**[b]	Phases[c]	*n*	*M*	*SD*	*t*	*p*
Assessment	1st<br>1st & 2nd	290<br>73	3.31<br>3.22	0.67<br>0.56	-1.13	.261
Psychoeducation	1st<br>1st & 2nd	290<br>73	3.24<br>3.33	0.65<br>0.65	-0.97	.335
Pharmacotherapy[d]	1st<br>1st & 2nd	217<br>30	3.17<br>3.19	0.39<br>0.37	-0.19	.853
Psychotherapy[d]	1st<br>1st & 2nd	263<br>25	2.48<br>2.69	0.69<br>0.69	-1.43	.155
**Components of Indication**[e]		*n*	*mean rank*			*p*
Primary Indication for Psychotherapy	1st<br>1st & 2nd	290<br>73	184.29<br>172.90			.393
Primary Indication for Pharmacotherapy	1st<br>1st & 2nd	290<br>73	178.97<br>194.05			.255
Indication for Pharmacotherapy prior to Psychotherapy	1st<br>1st & 2nd	290<br>73	182.06<br>181.76			.982
Indication for Psychotherapy prior to Pharmacotherapy	1st<br>1st & 2nd	290<br>73	180.09<br>189.60			.471

[a]HCPs = Health Care Providers
[b]Guideline Adherence Scores: means based on response options between "0" (almost never) and "4" (in up to 100% of cases)
[c]1st phase: global report about ADHD routine care via interview, 2nd phase: individual-based documentation of ADHD routine care
[d]Varying *n* : 1st due to branching logic in the interview whereas in 2nd phase, only those HCPs who applied interventions personally documented process
[e]As components of Treatment Indication exclude each other, comparisons were conducted item-wise via Mann-Whitney U test

Supplementary table S8

Comparison of GA indices[1] within each subsumed pair of professional groups

		Paediatricians[b] (*n*=25)					Psychiatrists[c] (*n*=26)					Psychotherapists[d] (*n*=22)				
**GA Indices[a]**	Pairs	*n*	*M*	*SD*	*t*	*p*	*n*	*M*	*SD*	*t*	*p*	*n*	*M*	*SD*	*t*	*p*
Assessment	Outpatient Units	8	69.14	12.80	1.78	.088	6	59.38	21.83	0.24	.814	16	51.46	22.09	-1.68	.108
	Practices	17	56.83	17.37			20	57.80	11.43			6	67.71	12.76		
Treatment Indication$_{OLD}$	Outpatient Units	8	80.83	13.89	-0.05	.960	6	84.72	15.29	0.06	.950	16	85.07	14.62	0.23	.819
	Practices	17	81.31	24.73			20	84.26	15.87			6	83.33	18.26		
Treatment Indication$_{NEW}$	Outpatient Units	8	95.83	11.79	0.78	.444	6	100.00	00.00	0.66	.514	16	100.00	00.00	n/a	n/a
	Practices	17	90.20	18.69			20	95.83	15.17			6	100.00	00.00		
Psychoeducation	Outpatient Units	8	14.24	13.07	0.22	.829	6	27.31	9.03	1.51	.144	16	24.68	13.15	-1.59	.128
	Practices	17	13.18	10.44			20	18.56	13.21			6	34.26	10.78		
Pharmacotherapy[5]	Outpatient Units	4	47.62	20.20	-0.63	.543	2	33.33	13.47	-2.04	.060	n/a	n/a	n/a	n/a	n/a
	Practices	9	53.62	13.95			15	52.70	12.56			n/a	n/a	n/a		
Psychotherapy[5]	Outpatient Units	n/a	n/a	n/a	n/a	n/a	2	20.83	5.89	-1.14	.372	15	37.87	13.07	-1.89	.075
	Practices	n/a	n/a	n/a			2	28.29	7.13			6	50.00	13.94		

[a]Guideline Adherence Indices: mean percentage of components fulfilled according to documented care of patients as rated by research assistants
[b]Outpatient Units of Social Paediatric Centres vs. Paediatric Practices
[c]Outpatient Units of Departments of Child and Adolescent Psychiatry vs. Child and Adolescent Psychiatric Practices
[d]Outpatient Units of Schools for Child and Adolescent Psychotherapy vs. Child and Adolescent Psychotherapeutic Practices
[e]Varying *n* as only Health Care Providers who applied these interventions personally documented process
[n/a]Not available

Supplementary table S9

Treatment characteristics of all *n*=167 patients

Interval-scaled variables	*M*	*SD*	*range*	*F*[a]	*p*
**Office visits during 6 months (contacts)**	**11.28**	**7.64**	**1.00 - 35.00**		
Paediatricians (*n*=50 patients)[1]	8.20	5.03	1.00 - 20.00		
Psychiatrists (*n*=81 patients)[1]	9.96	5.98	2.00 - 33.00	28.38	≤.001
Psychotherapists (*n*=36 patients)[2]	18.50	9.41	4.00 - 35.00		
**Duration of office visits (minutes)**	**53.51**	**16.88**	**10.00 - 132.50**		
Paediatricians (*n*=50 patients)[1]	44.05	19.98	10.00 - 83.08		
Psychiatrists (*n*=81 patients)[2]	57.07	14.04	34.09 - 132.25	12.50	≤.001
Psychotherapists (*n*=36 patients)[2]	58.36	12.92	50.00 -100.00		
**Duration of assessment (minutes)**	**261.37**	**199.74**	**30.00-1020.00**		
Paediatricians (*n*=50 patients)[1,2]	228.64	179.39	40.00-1020.00		
Psychiatrists (*n*=81 patients)[1]	303.01	231.34	30.00 - 960.00	3.35	.038
Psychotherapists (*n*=36 patients)[2]	205.71	98.71	50.00 - 450.00		
Dichotomous Variables	*no.*	*%*			
**Patients pre-treated by other clinicians (yes):**	**46**	**27.54**			
Paediatricians (*n*=50 patients)	7	14.00			
Psychiatrists (*n*=81 patients)	16	19.75			
Psychotherapists (*n*=36 patients)	23	63.59			
**Treatment will be continued (yes):**	**140**	**83.83**			
Paediatricians (*n*=50 patients)	42	84.00			
Psychiatrists (*n*=81 patients)	70	86.42			
Psychotherapists (*n*=36 patients)	28	77.77			

[a]Group differences were tested only for variables suitable for analyses of variance including post-hoc tests
[1,2]Groups with different numbers in index differ significantly (at least $p \leq .05$) in post-hoc test (Games-Howell)

Supplementary table S10

Correlations between globally reported (interview) and documented (rated protocols) guideline adherence of HCPs[a] participating in both phases (1st & 2nd)

			Rated Protocols
**Interview**	Components[b]		
Assessment (AS)	Mandatory	*r*	.416***
		(n)	(73)
	All	*r*	.522***
		(n)	(73)
Psychoeducation (PE)	Mandatory	*r*	.045
		(n)	(73)
	All	*r*	.226
		(n)	(73)
Pharmacotherapy (PH)	Mandatory	*r*	.108
		(n)	(30)[c]
	All	*r*	.084
		(n)	(30)[c]
Psychotherapy (PT)	Mandatory	*r*	-.097
		(n)	(25)[d]
	All	*r*	.296
		(n)	(25)[d]

[a]HCPs = Health Care Providers
[b]Detailed information about specific components included in All vs. Mandatory can be found in Tables S1-S5; as components of Treatment Indication in the interview exclude each other, correlations could not be analysed
[c]Results refer to *n*=13 paediatricians and *n*=17 psychiatrists who treated their patients personally
[d]Results refer to *n*=4 psychiatrists and *n*=21 psychotherapists who treated their patients personally
***p ≤ .001 (2-tailed) **p ≤ .01 (2-tailed) *p ≤ .05 (2-tailed)
